# Two-dimensional data storage using a DNA molecular register

**DOI:** 10.3389/fbioe.2026.1927944

**Published:** 2026-09-11

**Authors:** Sirui Li, Pali Ye, Zhen Xiong, Junkeng Huang, Hao Guan, Xiaoli Qiang, Xiaolong Shi

**Affiliations:** 1 Institute of Computing Science and Technology, Guangzhou University, Guangzhou, China; 2 Department of Materials Science and Engineering, City University of Hong Kong, Hong Kong, China; 3 School of Computer Science and Cyber Engineering, Guangzhou University, Guangzhou, China

**Keywords:** coordinate addressing, DNA nanotechnology, DNA origami, molecular register, two-dimensional data storage

## Abstract

Existing molecular data-storage systems still mainly encode information along one-dimensional chemical sequences or linear molecular coordinates, leaving the spatial addressability of molecular architectures insufficiently explored. Here we report a physical two-dimensional DNA molecular register, termed a spatially addressable molecular breadboard (SAMB), in which digital bits are assigned to predefined molecular coordinates rather than to positions along a one-dimensional sequence. SAMB is built by hierarchical assembly of DNA origami single modules into a globally registered two-dimensional address space. In this register, each data or parity bit is encoded by the presence or absence of streptavidin at a defined coordinate site and read out from local topographic height differences, whereas the fiducial marker (FM) site defines register orientation and coordinate alignment. As a proof of concept, we encode a Petersen graph payload in a 49-coordinate SAMB register and demonstrate *in situ* atomic force microscopy (AFM) readout, coordinate-based bit calling, and graph reconstruction. We further incorporate three block-wise even-parity positions as proof-of-concept design elements. In a randomly selected test subset of 56 readable SAMB structures, the 45 graph-bit positions yielded a raw graph-bit accuracy of 86.79%, with false-positive and false-negative rates of 7.32% and 25.00%, respectively; the three parity-bit positions, evaluated separately, yielded a raw site-readout accuracy of 82.14%. These results show that DNA nanostructures can serve as physical two-dimensional molecular registers for coordinate-addressed data storage and provide a basis for topography-readable molecular information systems.

## 1 Introduction

DNA has been widely explored as a next-generation medium for digital information storage because of its ultrahigh information density, long-term chemical stability and sequence programmability1-7. In most existing DNA storage systems, digital information is encoded into the linear order of A, G, C and T nucleotides and is recovered by DNA sequencing followed by computational decoding of sequence reads (Wang et al., 2024; Yu et al., 2024; Doricchi et al., 2022; Jo et al., 2024). This sequence-centered paradigm has enabled remarkable advances in storage density, archival stability and coding efficiency (Gao and No, 2024; Huang et al., 2025; Weindel et al., 2025). However, the information is still physically organized as a one-dimensional polymeric code, and reliable data recovery depends on accurate synthesis, amplification, sequencing, alignment and consensus reconstruction (Song et al., 2022; Welzel et al., 2023). Insertions, deletions, substitutions and strand loss introduced during synthesis, storage or sequencing can therefore disrupt sequence interpretation and require indexing, redundancy and error-correction codes for robust decoding (Yu et al., 2024; Welzel et al., 2023; Ding et al., 2024).

Moving beyond one-dimensional sequence encoding, spatially organized DNA systems offer an alternative route for storing information in molecular space (Li et al., 2026; Jiang et al., 2024). In such systems, information can be associated with predefined molecular positions rather than solely with nucleotide order, allowing data states to be interpreted according to addressable spatial locations (Doricchi et al., 2022; Pan et al., 2022; Dickinson et al., 2021). Representative systems illustrate several distinct routes beyond conventional sequence-only storage. Non-sequence nucleic acid memory placed addressable docking-strand states on individual DNA origami and recovered them using DNA points accumulation for imaging in nanoscale topography (DNA-PAINT); rewritable two-dimensional DNA storage distributed information between nucleotide sequences and backbone nicks and relied on sequencing-assisted reconstruction; DNA origami cryptography used spatial protein or fluorescent patterns, orientation markers, and atomic force microscopy (AFM) or super-resolution microscopy for secure message recovery; and linked DNA-origami storage used shape-defined nodes, local streptavidin occupancy, and strand-programmed pointers to support data insertion and deletion (Pan et al., 2022; Zhang et al., 2025; Dickinson et al., 2021; Wisna et al., 2025; Strauss et al., 2018; Zhang et al., 2019). These studies establish important precedents for spatial labeling, orientation-assisted decoding, and structure-based data representation. Distinct from these prior systems, the present design integrates four cross-shaped DNA origami modules into a single globally registered 49-coordinate physical register, in which a common fiducial marker establishes the global coordinate frame and liquid-phase AFM enables direct address-resolved topographic readout. A detailed feature-by-feature comparison with the present design is provided in Supplementary Table S1.

DNA origami provides a particularly suitable platform for constructing such coordinate-addressed molecular registers (Wang et al., 2025; Weck and Heuer-Jungemann, 2025). Its programmable geometry, nanoscale addressability and structural modularity allow molecular features to be positioned with defined spatial relationships (Pan et al., 2022; Dickinson et al., 2021; Dey et al., 2021; Zhou et al., 2023). In principle, each predefined address on a DNA origami scaffold can serve as a molecular bit position, and the local occupancy state of a molecular label at that address can be used to represent a binary state (Rabbe et al., 2025; Weck and Heuer-Jungemann, 2025). AFM can resolve DNA origami geometry and molecular height differences at the nanoscale, making it possible to read topographic occupancy patterns directly from individual nanostructures (Zhang et al., 2025; Rabbe et al., 2025; Kolbeck et al., 2023). In addition, fiducial markers on DNA origami can provide spatial references for orientation registration and coordinate alignment, which are essential for address-level decoding (Zhang et al., 2025; Kolbeck et al., 2023).

Recent advances have further demonstrated the potential of DNA-based molecular logic for error detection and information security. Mercury-mediated DNA–Au/Ag nanocluster ensembles have been used to implement parity generation and checking together with binary-to-Gray-code conversion, illustrating the integration of molecular computation with error-aware information encoding (Mattath et al., 2022). Subsequently, DNA-templated nanoclusters bearing specific overhangs enabled the construction of contrary logic pairs and parity-based detection of errors in binary transmission (Mattath et al., 2023). More recently, a molecular dye–oligonucleotide framework integrating Morse code, logic based on the American Standard Code for Information Interchange (ASCII), and Beale’s cipher established a multilevel strategy for molecular crypto-steganography (Mattath et al., 2026). These studies demonstrate the utility of nucleic-acid-based molecular logic for error-aware encoding and information security, but primarily focus on biochemical logic operations and cryptographic transformations rather than direct information representation at predefined spatial addresses.

Beyond nucleic-acid-based systems, biogenic fluorescent carbon dots provide a chemically responsive approach to molecular information processing. Parambil et al. used bulk, thiol-functionalized, and acid-functionalized carbon-dot platforms to construct a concatenated logic library, an even/odd parity checker, and a pattern-mediated molecular keypad lock through stimulus-dependent photoluminescence (Parambil et al., 2023). In such systems, information is represented by chemically modulated optical input–output states, enabling rapid fluorescence readout and offering potential applications in chemical sensing, responsive molecular logic, and information security. However, their extension toward durable, high-capacity data storage would require reproducible multiplexing of orthogonal chemical inputs, stable calibration of photoluminescent outputs, and systematic evaluation of long-term retention and batch-to-batch variability. By contrast, SAMB encodes information through streptavidin occupancy at predefined coordinates within a four-module DNA origami architecture, providing explicit spatial addressing, a common orientation reference, and address-level topographic readout and error mapping. These capabilities come at the cost of multistep assembly and comparatively low-throughput AFM imaging. Carbon-dot and SAMB platforms therefore serve complementary roles: the former are particularly suited to stimulus-responsive sensing and molecular logic, whereas the latter are designed for spatially addressable physical data representation.

Here, we report a proof-of-concept two-dimensional DNA molecular register, termed a spatially addressable molecular breadboard (SAMB), that stores digital information as molecular occupancy states within a physical two-dimensional coordinate space. SAMB is assembled from four cross-shaped DNA origami single modules (SMs) into a tic-tac-toe-like architecture, forming a globally registered molecular address space. Each bit is assigned to a predefined coordinate and encoded by the absence or presence of streptavidin at that address. A fiducial marker (FM) is incorporated to break rotational symmetry, define the global orientation and establish the coordinate origin, enabling all addresses to be interpreted within a unified reference frame.

As a model payload, we encoded a Petersen graph in a 49-coordinate SAMB register, comprising 45 graph bits derived from the upper-triangular adjacency matrix, 3 parity bits and one FM site. The Petersen graph was selected because it is a compact classical graph with non-trivial topology, allowing relational graph information to be converted into a spatial molecular occupancy pattern. Using liquid-phase AFM, we demonstrate coordinate-based bit calling, graph-bit extraction and reconstruction of the target graph from a representative SAMB register. We further evaluate the readout output across 100 SAMB registers by quantifying raw bit accuracy, false-positive and false-negative events, and address-level error distributions. Finally, we examine the compatibility of the SAMB design with block-wise parity validation and erasure-aware output assessment. Together, these results demonstrate that DNA nanostructures can serve as physical two-dimensional molecular registers for coordinate-addressed data storage and direct topographic readout.

## 2 Materials and methods

### 2.1 DNA origami design

The cross-shaped single module (SM) was designed using DNA origami principles and folded from M13mp18 scaffold DNA and a set of staple strands. Each arm of the SM was designed as a six-helix bundle (6HB), providing a mechanically stable framework for positioning molecular addresses in two-dimensional space. Predefined address sites were arranged along the SM arms for streptavidin-mediated bit encoding. Addresses designed as bit 1 contained biotin-modified staple strands for streptavidin binding, whereas addresses designed as bit 0 did not contain the corresponding biotin modification. The complete staple sequences and chemical modifications are listed in Supplementary Table S2.

### 2.2 Folding of single modules

SMs were folded by mixing M13mp18 scaffold DNA with the corresponding staple strands at a molar ratio of 1:5. Folding reactions were carried out in buffer containing 1 × TAE and 12.5 mM ${\text{MgCl}}_{2}$ . The mixture was annealed from 95 °C to 4 °C, with the temperature decreased in increments of 0.1 °C every 40 s. Folded products were purified using 100 k Da centrifugal filters to remove excess staple strands and stored at 4 °C in 1 × TAE buffer containing 12.5 mM ${\text{MgCl}}_{2}$ until use.

### 2.3 Assembly of SAMB registers

The spatially addressable molecular breadboard (SAMB) was assembled from four cross-shaped SMs through sequence-specific sticky-end interactions at the module edges. SM1–SM4 were mixed at a molar ratio of 1:1:1:1 in assembly buffer containing 1 × TAE and 12.5 mM ${\text{MgCl}}_{2}$ . The mixture was incubated from 45 °C to 20 °C over 13 h to form a 2×2 tic-tac-toe-like DNA origami array. An asymmetric fiducial marker (FM) was placed on SM1 to break rotational ambiguity and define the global orientation and coordinate origin of the SAMB register.

### 2.4 Streptavidin loading and bit encoding

Binary information was encoded by the presence or absence of streptavidin (SA) at predefined molecular addresses. The SAMB samples were incubated with 100 nM streptavidin in 1 × PBS for 2 h. Addresses designed as bit 0 lacked the corresponding biotin modification. After incubation, excess streptavidin was removed by centrifugal filtration. In the final register, SA-absent addresses were assigned as bit 0, and SA-occupied addresses were assigned as bit 1.

### 2.5 Petersen graph payload design and coordinate mapping

The target payload was derived from the Petersen graph. The upper-triangular elements of its 10×10 adjacency matrix were extracted to generate 45 graph bits. Together with 3 parity bits and one FM site, these positions formed a 49-coordinate SAMB register. The FM site was used for orientation registration and coordinate alignment, whereas the remaining 48 non-FM data-bearing positions comprised 45 graph-payload bits and three parity bits. Each non-FM data-bearing position was assigned to a predefined molecular address within the four-module SAMB register. The complete coordinate mapping is provided in Supplementary Figure S1 and Supplementary Tables S3, S4.

### 2.6 Liquid-phase AFM imaging

For AFM sample preparation, 20 μ L of a 2.5 nM SAMB solution was deposited onto freshly cleaved mica and allowed to adsorb for 20 min at room temperature. The mica was then rinsed three to five times with ultrapure water in a Petri dish to remove unbound structures. The sample was subsequently covered with imaging buffer containing 1 × TAE and 12.5 mM ${\text{MgCl}}_{2}$ for liquid-phase AFM imaging at room temperature. AFM images were acquired using either a MultiMode 8 atomic force microscope (Bruker) or a Cypher ES atomic force microscope (Asylum Research, Oxford Instruments). The MultiMode 8 was operated in ScanAsyst/PeakForce Tapping mode using a SCANASYST-FLUID+ probe comprising a silicon tip on a silicon nitride cantilever. The probe had a nominal spring constant of 0.7 N ${\text{m}}^{-1}$ and a nominal resonant frequency of 150 kHz. Images were acquired at a scan rate of 0.988 Hz and a resolution of up to 512×512 pixels. The PeakForce setpoint, PeakForce amplitude, and PeakForce frequency were 0.1000 V, 100 nm, and 2 kHz, respectively, and the feedback gain was 2.644. The Cypher ES was operated in liquid-phase AC mode using an Olympus BL-AC40TS-C2 cantilever with a nominal spring constant of 0.09 N ${\text{m}}^{-1}$ and a nominal resonant frequency of 110 kHz. Images were acquired at a scan rate of 1.0 Hz and a resolution of 256×256 pixels. The amplitude setpoint was 240 mV, and the feedback gain was 30. For both AFM systems, scan sizes ranged from 500 nm×500 nm to 2 μm×2 μm . All images used for address-level height extraction and binary bit calling were acquired under the corresponding liquid-phase imaging conditions described above.

### 2.7 Coordinate registration and address-level height extraction

AFM images were processed using a custom analysis workflow. Individual SAMB registers were first identified from AFM fields of view. Molecules with complete four-module structures were retained, whereas registers showing severe folding, incomplete assembly or substantial global deformation were excluded. For each retained register, the FM was used to determine the global orientation and coordinate origin. The predefined address map was then overlaid onto the AFM height image. For each predefined address $P_{i}$ , a spatial mask $M_{i}$ was applied around the expected coordinate position. The local height $h_{i}$ was defined as the local peak height within $M_{i}$ after correction against the neighboring DNA origami scaffold background. Regions outside the mask were not used for bit calling.

### 2.8 Binary bit calling

Binary bit values were assigned using a fixed AFM height threshold. The bare 6HB DNA origami scaffold showed a height of approximately 2 nm, whereas streptavidin-bound addresses produced higher local topographic features. Based on height measurements from 300 predefined address sites, 3.7 nm was selected as the decision threshold for binary bit calling. Addresses with $h_{i}<3.7$ nm were assigned as bit 0, whereas addresses with $h_{i}\geq 3.7$ nm were assigned as bit 1. This workflow generated an AFM-called register output for each analyzed SAMB molecule, including one FM site and 48 non-FM data-bearing positions.

### 2.9 Parity-block definition, erasure classification, and decoding

Each SAMB register contained one fiducial marker (FM) for orientation and coordinate registration and 48 non-FM data-bearing positions. The non-FM positions comprised 45 Petersen-graph bits and three even-parity bits. The FM was excluded from parity checking, erasure counting, and graph-payload recovery analysis. Let $x_{i}\in \left\{0,1\right\}$ denote the binary state at position $P_{i}$ . Three non-overlapping even-parity blocks were defined as

$$\begin{array}{cc} B_{1} & =\left\{P_{1},\ldots ,P_{10},P_{12}\right\},\\ B_{2} & =\left\{P_{11},P_{13},\ldots ,P_{36},P_{47}\right\},\\ B_{3} & =\left\{P_{37},\ldots ,P_{46},P_{48}\right\}. \end{array}$$

where $P_{12}$ , $P_{47}$ , and $P_{48}$ were the parity positions of the three blocks, respectively. Each block satisfied

$$\underset{i\in B_{k}}{⊕}x_{i}=0,\;k=1,2,3,$$

where ⊕ denotes the exclusive-OR operation.

The height thresholds and decoding rules were fixed before independent testing. Let $h_{i}$ denote the locally background-corrected AFM height at position $P_{i}$ . Each position was classified according to

$${\tilde{x}}_{i}=\left\{\begin{array}{cc} 0, & h_{i}\leq 3.64\;nm,\\ ?, & 3.64\;nm<h_{i}<3.76\;nm,\\ 1, & h_{i}\geq 3.76\;nm. \end{array}\right.$$

where “?” denotes an erasure. Thus, an ambiguous signal was classified as an erasure when

$$\left|h_{i}-3.70\;nm\right|<0.06\;nm.$$

The number of erasures, $E_{k}$ , was determined separately for each parity block. When $E_{k}=0$ , even parity was checked directly, and a parity failure caused the block to be rejected. When $E_{k}=1$ and the unique erased position was e , its value was inferred as

$${\hat{x}}_{e}=\underset{\begin{array}{c} j\in B_{k}\\ j\neq e \end{array}}{⊕}{\hat{x}}_{j}.$$

The inferred value was then inserted, thereby restoring the even-parity relation by construction. This procedure could recover a single erasure at a known position but did not provide an independent means of detecting an unflagged hard-decision error within the same block. When $E_{k}\geq 2$ , the block was considered undecodable because one parity equation cannot uniquely determine multiple erased bits. Under erasure-aware parity decoding, a register was accepted only when all three blocks contained no remaining erasures and satisfied their respective parity constraints.

Four approaches were compared using a prespecified independent test set of 56 registers: (A) hard-decision calling at 3.70 nm , which produced an output for every register; (B) parity-only validation, which accepted a register only if all three blocks satisfied even parity; (C) erasure-only rejection, which accepted a register only if none of its 48 non-FM positions was classified as an erasure; and (D) erasure-aware parity decoding according to the rules described above. Rejected or undecodable registers were counted as recovery failures. Because each register encoded one Petersen-graph payload, strict graph-payload recovery and strict graph recovery were equivalent.

### 2.10 Graph reconstruction

For each analyzed SAMB register, the AFM-called register output was parsed into one FM site, 3 parity bits, and 45 graph bits. The 45 graph bits were mapped to the upper-triangular elements of a 10×10 adjacency matrix. The lower-triangular elements were filled by symmetry, and the diagonal elements were set to 0. The resulting adjacency matrix was used to reconstruct the Petersen graph.

### 2.11 Population-level readout analysis

Population-level readout performance was evaluated from SAMB candidate molecules identified in liquid-phase AFM images. Candidate registers were screened for four-module structural completeness, absence of severe folding or global deformation, and identifiable FM signals. Structurally intact SAMB registers with successful FM-based coordinate registration were included in the analysis. For each retained register, raw bit accuracy was calculated before parity-based validation or erasure-aware assessment. Because the FM was used to define register orientation and coordinate alignment, it was not included as The FM in the accuracy analysis. Raw bit accuracy was therefore calculated from was excluded from the accuracy analysis. Raw bit accuracy was therefore calculated over 48 non-FM evaluated positions, comprising 45 graph-payload bits and three parity bits. False-positive and false-negative rates were also calculated from these 48 non-FM bit calls. Address-level error frequency was determined by comparing the AFM-called bit value with the designed bit state at each predefined non-FM molecular coordinate across the analyzed SAMB registers.

### 2.12 Statistical analysis

AFM images were processed using Gwyddion software, including image flattening, background correction, cross-sectional height analysis and address-level height extraction. Local peak heights used for bit calling were extracted from predefined molecular addresses after local background correction. Raw bit accuracy, false-positive and false-negative rates, and address-level error frequencies were calculated from raw bit calls. Statistical analysis and graph generation were performed using GraphPad Prism. Unless otherwise stated, data are presented as mean ± s.d. or as box plots. Box plots show the median and interquartile range, as described in the corresponding figure legends.

## 3 Results

### 3.1 SAMB establishes a stable two-dimensional molecular address space

**FIGURE 1:**
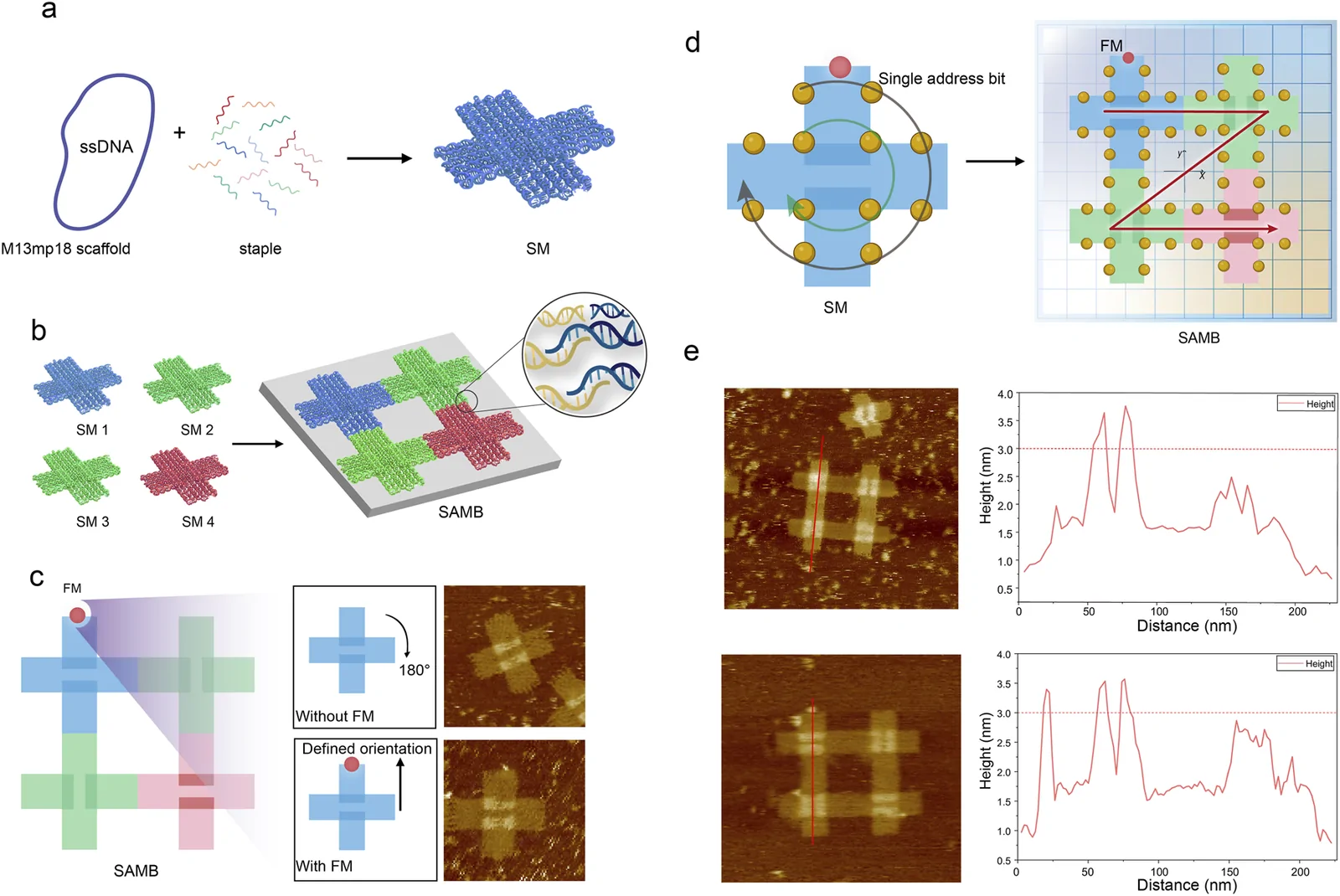
Architectural design and coordinate registration of the SAMB molecular register. **(a)** Design and assembly of the cross-shaped DNA origami single module (SM). Each SM is folded from the M13mp18 scaffold strand and staple strands, with each arm designed as a six-helix bundle (6HB) structure. **(b)** Assembly of the 2×2 SAMB. Four SMs (SM1–SM4) are connected through sequence-specific sticky-end hybridization to form a two-dimensional DNA origami array. **(c)** FM-mediated orientation registration. The fiducial marker (FM) on SM1 breaks rotational ambiguity and defines the global orientation and coordinate origin. **(d)** Coordinate-addressed bit readout path. Non-FM molecular addresses are queried in a predefined order across SM1–SM4 and converted into an ordered digital output according to streptavidin occupancy. **(e)** AFM height calibration. Height profiles resolve the topographic difference between the DNA origami background and streptavidin-bound addresses, providing the basis for subsequent bit 0/1 calling.

We designed SAMB as a modular DNA origami register in which molecular addresses are fixed in a two-dimensional layout. The basic unit is a cross-shaped DNA origami single module (SM), folded from the M13mp18 scaffold strand and a set of staple strands (Figure 1a). Each arm of the SM was built as a six-helix bundle (6HB), providing a rigid nanoscale support for address positioning and helping to limit structural deformation during liquid-phase AFM imaging.

A complete SAMB register was assembled from four independent SMs. Each connecting edge of an SM carried six sticky ends, allowing neighboring modules to hybridize through sequence-specific interactions. This hierarchical assembly generated a 2×2 tic-tac-toe-like DNA origami array composed of SM1–SM4 (Figure 1b). In this architecture, the four SMs together define a continuous two-dimensional molecular address space for coordinate-based encoding and readout.

Because the four-module SAMB structure is approximately rotationally symmetric, reliable decoding requires a fixed orientation. To remove this ambiguity, an asymmetric fiducial marker (FM) was placed on SM1 (Figure 1c). The FM breaks rotational symmetry, defines the coordinate origin and allows each register to be aligned to the same coordinate frame. Representative AFM images confirmed that the FM could be identified in assembled SAMB structures, enabling the designed address map to be overlaid onto individual registers before bit calling.

Within the FM-defined coordinate system, each non-FM molecular address was assigned to a graph or parity bit, whereas the FM site was used for orientation registration. The binary state of each non-FM address was encoded by streptavidin (SA) occupancy: addresses without SA were defined as bit 0, and SA-occupied addresses were defined as bit 1 (Figure 1d). By querying the addresses in a predefined order across SM1–SM4, the two-dimensional molecular occupancy pattern could be converted into an ordered digital output while retaining the physical coordinate identity of each bit.

We then calibrated the height contrast needed for address-level readout using liquid-phase AFM (Figure 1e). The bare 6HB DNA origami scaffold showed a background height of approximately 2 nm, whereas SA-bound addresses appeared as higher local features. Height measurements from 300 predefined address sites were used to set a fixed 3.7 nm threshold for binary bit calling. Neighboring addresses were spaced by more than 10 nm, which allowed local height extraction at individual sites and reduced signal overlap between adjacent addresses.

Together, the four-module origami architecture, FM-based registration and AFM-resolved height contrast define SAMB as a physical two-dimensional DNA molecular register for coordinate-addressed bit encoding and readout.

### 3.2 Two-dimensional coordinate mapping of the Petersen graph payload

**FIGURE 2:**
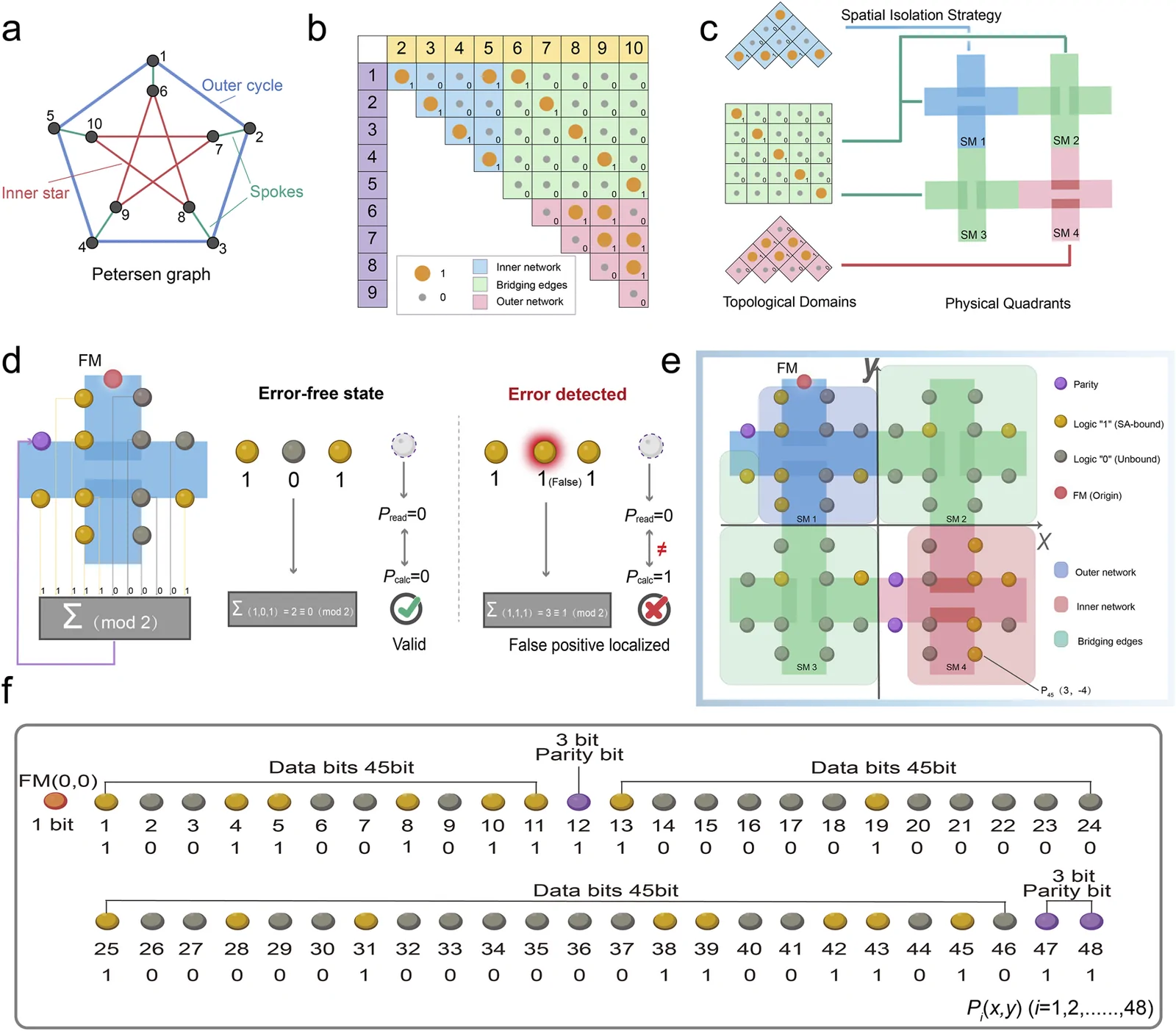
Physical two-dimensional molecular data encoding based on a Petersen graph payload. **(a)** Petersen graph used as the model payload, consisting of three classes of connections: the outer cycle, inner star and spokes. **(b)** Upper-triangular adjacency matrix of the Petersen graph. The 45 matrix elements are defined as graph bits, with colors indicating inner-network, bridging-edge and outer-network connections. **(c)** Mapping of graph topology onto the physical domains of SAMB. Distinct topological domains are assigned to corresponding spatial regions across SM1–SM4. **(d)** Schematic of parity-based validation. The streptavidin occupancy state defines the bit value, and parity calculation is used to detect local readout inconsistencies. **(e)** Spatial distribution of the 49-coordinate SAMB register within the global coordinate grid. The FM defines the coordinate origin and global orientation. **(f)** Serialized layout of the 49-coordinate SAMB register, including one FM site, 45 graph-bit positions and 3 parity-bit positions, with each bit assigned to a predefined coordinate $P_{i}\left(x,y\right)$ .

To test whether SAMB can encode structured digital information in a two-dimensional molecular coordinate space, we designed a 49-coordinate register containing 45 graph bits, 3 parity bits and one FM site (Figures 2a,b). The 45 graph bits were derived from the upper-triangular elements of the 10×10 adjacency matrix of the Petersen graph, in which each matrix element represents the presence or absence of an edge between a pair of graph nodes. The remaining positions included three parity bits for block-level validation and one FM site for orientation registration.

Each non-FM data-bearing position was assigned to a predefined molecular address within the four-module SAMB register. To reduce potential readout interference between neighboring occupied sites, the graph bits were distributed across the spatial domains of SM1–SM4 and further organized according to the topological classes of the Petersen graph, including the outer cycle, inner star and bridging edges (Figure 2c). This mapping strategy preserves the logical structure of the graph while increasing spatial separation between selected SA-occupied addresses, thereby reducing the potential effects of AFM tip broadening, local steric crowding and signal overlap on bit calling.

The three parity bits were incorporated as simple block-wise validation constraints rather than as a complete error-correction system (Figure 2d). Each parity bit was calculated from a predefined block of graph-payload bits and was used to evaluate whether the decoded output remained consistent with the designed register logic. Thus, the parity design provides a lightweight means of detecting local inconsistencies or flagging unresolved regions when ambiguous address-level signals are treated as erasures.

The final 49-coordinate design was mapped onto the global SAMB coordinate grid defined by the FM (Figures 2e,f). In this design, each bit has both a logical identity, defined by its position in the serialized register output, and a physical identity, defined by its coordinate within the two-dimensional molecular register. This coordinate-defined mapping establishes the interface between relational graph information and physical molecular occupancy, providing the basis for AFM-based readout and graph reconstruction.

### 3.3 AFM-based coordinate readout and graph reconstruction from a representative SAMB register

**FIGURE 3:**
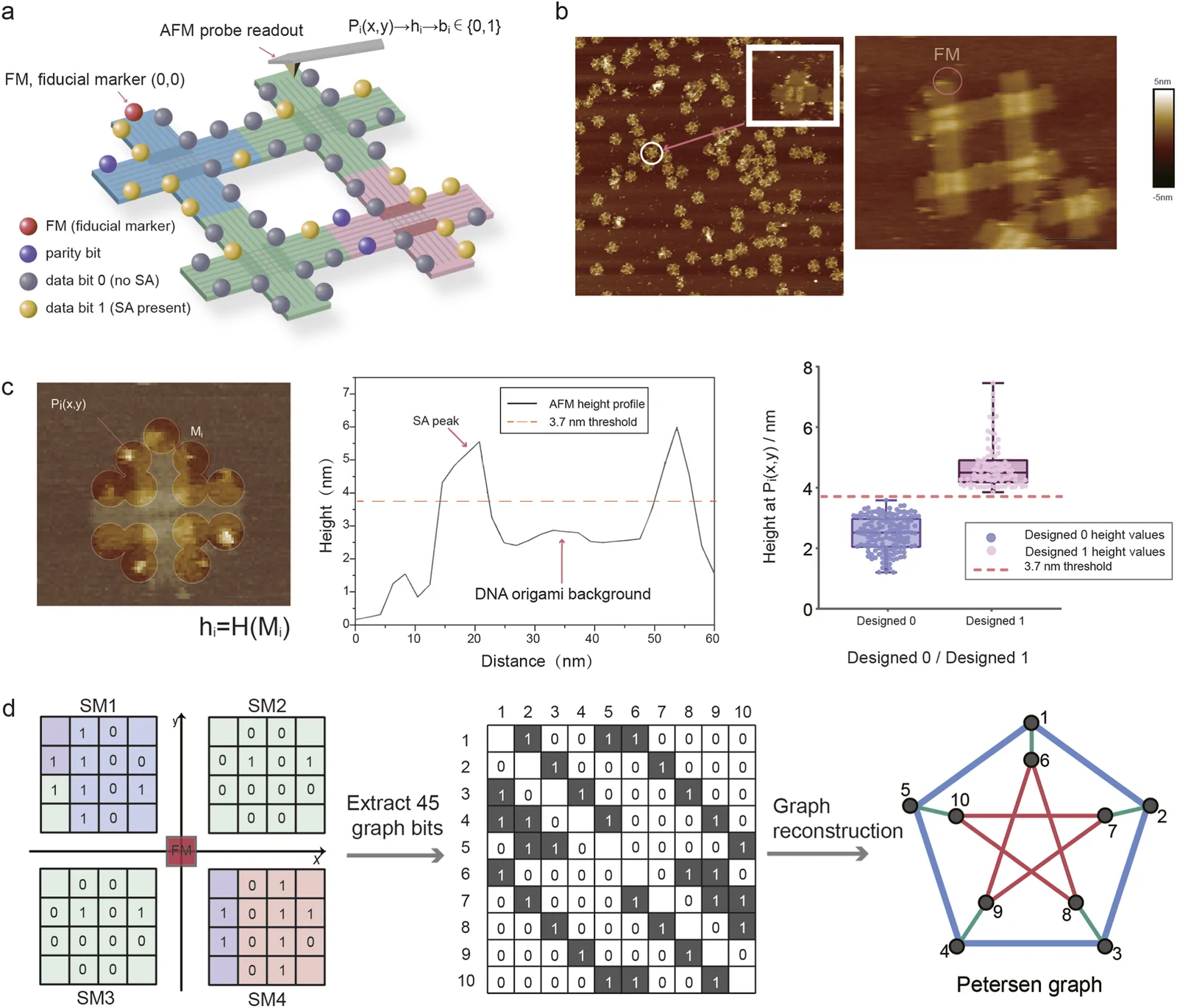
Coordinate-addressed AFM readout and graph reconstruction from a representative SAMB register. **(a)** Schematic of coordinate-addressed AFM readout of the SAMB register. Each predefined address was assigned a binary value according to the local streptavidin occupancy state, generating a serialized output from the 49-coordinate register. The FM defines the coordinate origin and global orientation. **(b)** Representative liquid-phase AFM images. The large-area image shows SAMB candidate structures, and the magnified image shows a SAMB register with an identifiable FM. Scale bars, 100 nm. **(c)** Address-level height extraction and bit calling. The left panel shows predefined addresses $P_{i}$ and spatial masks $M_{i}$ used for local height extraction. The local height was defined as $h_{i}=H\left(M_{i}\right)$ . The middle panel shows a representative height profile, with the dashed line indicating the 3.7 nm decision threshold. The right panel shows the height distributions of designed 0 and designed 1 addresses. Scale bar, 50 nm. **(d)** Extraction of 45 graph bits from the AFM-called register output, mapping to the upper-triangular adjacency matrix, and reconstruction of the Petersen graph from the decoded matrix.

After defining the SAMB architecture and payload map, we asked whether a complete 49-coordinate molecular register could be read directly from AFM images and decoded into the target graph. Rather than relying on global image-feature recognition, the readout was performed at predefined two-dimensional molecular addresses. At each address, liquid-phase AFM was used to measure the local topographic signal associated with streptavidin occupancy and convert it into a binary value (Figure 3a).

Representative AFM images showed multiple SAMB candidate structures. From these images, we selected one register with a complete four-module architecture and a clearly identifiable FM for coordinate-based decoding (Figure 3b). The FM was first used to determine the global orientation and coordinate origin. The designed address map was then overlaid onto the AFM height image, allowing each molecular address to be queried at its expected position. This procedure preserved the one-to-one relationship between bit identity and physical coordinate.

For address-level bit calling, a spatial mask $M_{i}$ was applied around each predefined address $P_{i}$ , and the local height $h_{i}$ was extracted from the AFM height map (Figure 3c). Local background correction was performed using the neighboring DNA origami scaffold. Addresses with $h_{i}<3.7$ nm were called as bit 0, whereas addresses with $h_{i}\geq 3.7$ nm were called as bit 1. This generated an AFM-called register output containing one FM site, 3 parity bits and 45 graph bits.

The 45 graph bits were then mapped back to the upper-triangular elements of a 10×10 adjacency matrix (Figure 3d). A value of 1 denotes an edge between the corresponding pair of graph nodes, whereas a value of 0 denotes no edge. The lower-triangular elements were filled by symmetry, and the diagonal elements were set to 0. The resulting adjacency matrix was used to reconstruct the Petersen graph.

In this representative register, the FM was clearly identified, and all 48 non-FM evaluated positions, comprising 45 graph-payload bits and three parity bits, were correctly called before parity-based validation. The decoded graph retained the designed Petersen topology, showing that a two-dimensional molecular occupancy pattern on SAMB can be converted into graph-level digital output through FM-guided coordinate registration, address-level height extraction and binary bit calling.

### 3.4 Population-level readout performance and address-level error distribution

To evaluate whether SAMB readout was reproducible beyond a single representative register, we performed population-level analysis using liquid-phase AFM images of multiple SAMB candidate molecules. Candidate structures were screened according to predefined criteria, including four-module structural completeness, absence of severe folding or global deformation, and FM recognizability. Finally, 100 structurally intact SAMB registers with identifiable FMs and successful coordinate registration were included in the bit-calling analysis.

**FIGURE 4:**
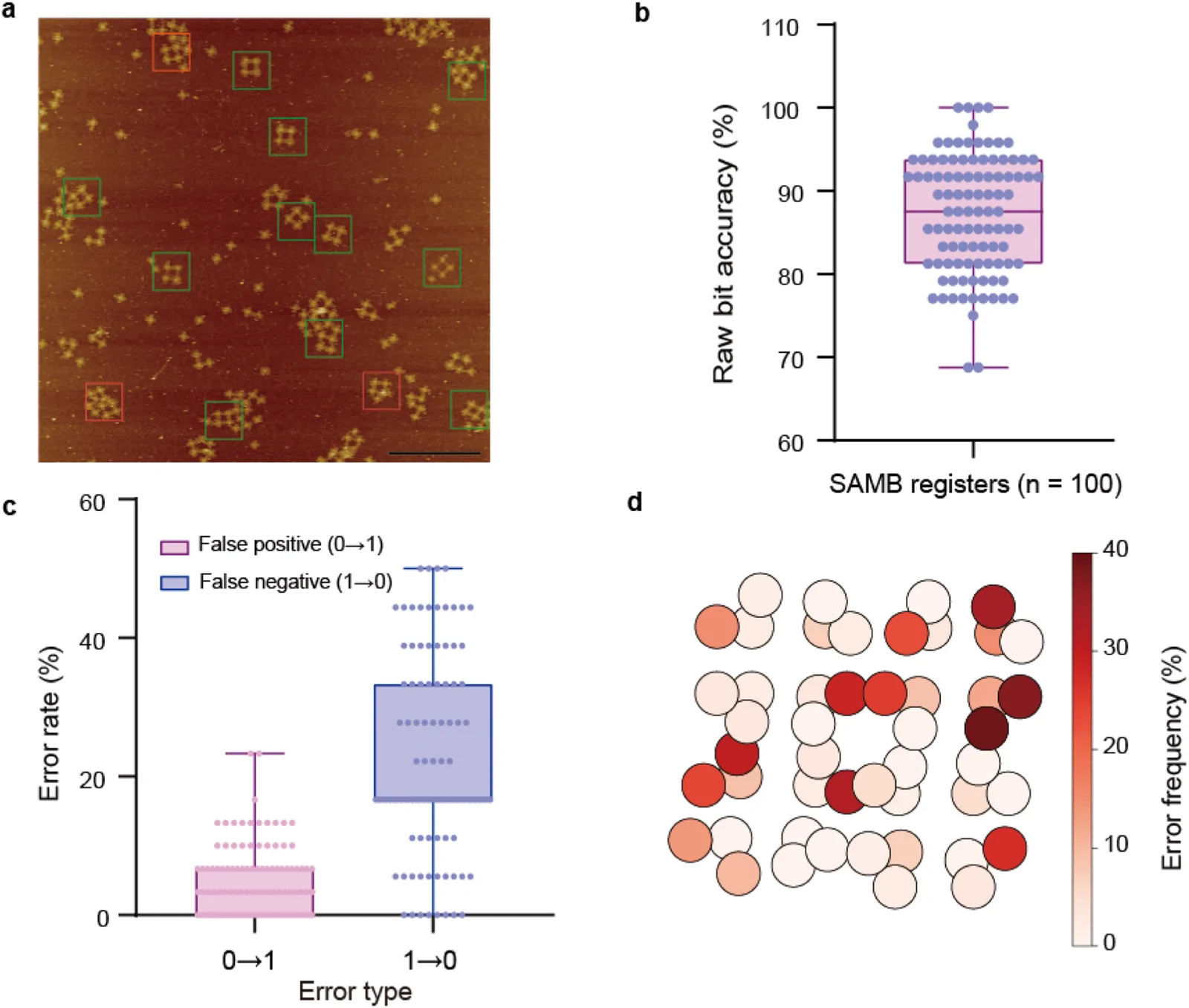
Population-level readout reliability of the two-dimensional SAMB registers. **(a)** Representative large-area AFM image used for population-level analysis. A total of 100 SAMB registers with completed coordinate registration were included in the analysis. Scale bar, 800 nm. **(b)** Raw bit accuracy of 100 SAMB registers, calculated from 48 non-FM evaluated positions, comprising 45 graph-payload bits and three parity bits. The FM site was excluded because it was used for orientation registration. Each point represents one register; boxes show the median and interquartile range. **(c)** False-positive and false-negative error rates in raw bit calling. A false positive indicates that an address designed as 0 was called as 1, whereas a false negative indicates that an address designed as 1 was called as 0. **(d)** Spatial error-frequency map of predefined non-FM SAMB addresses, $P_{i}\left(x,y\right)$ . Color indicates the frequency at which each address was miscalled relative to its designed bit state across the 100 registers.

For each analyzed register, we calculated raw bit accuracy before parity-based validation or erasure-aware assessment. Because the FM was used to define register orientation and coordinate alignment, it was excluded from the population-level accuracy analysis. Raw bit accuracy was therefore calculated over the 48 non-FM evaluated positions, comprising 45 graph-payload bits and three parity bits. Across the 100 SAMB registers, the raw bit accuracy was 87.0±7.1% (mean ± s.d.). Representative population-level AFM imaging and the distribution of raw bit accuracy across the 100 analyzed SAMB registers are shown in Figures 4a,b. These results show that the coordinate-based AFM readout can recover the designed molecular occupancy pattern across multiple SAMB registers, while also revealing address-level errors that require further analysis.

We next quantified the two major classes of binary readout errors: false-positive events, in which an address designed as 0 was called as 1, and false-negative events, in which an address designed as 1 was called as 0 (Figure 4c). False-positive events may arise from nonspecific adsorption, local background fluctuations or imaging artifacts, whereas false-negative events may result from incomplete streptavidin loading, loss of streptavidin occupancy or insufficient height contrast during AFM imaging. The false-negative rate was higher than the false-positive rate, suggesting that the dominant source of raw bit-calling error in the current SAMB system is more likely the loss or under-detection of SA occupancy signals rather than misclassification of empty sites. Although the present data do not distinguish among these mechanisms, this error profile provides guidance for future optimization of molecular label stability, address spacing and imaging conditions.

Because each non-FM evaluated position in SAMB is assigned to a known physical coordinate, readout errors can be mapped back to their corresponding molecular addresses. We therefore generated an address-level error-frequency map across the 100 analyzed registers (Figure 4d). The error distribution was not uniform across all predefined coordinates, indicating that local structural environment, molecular crowding, spatial occlusion or AFM imaging conditions may influence the readout stability of specific addresses. This spatially resolved error analysis is an important feature of coordinate-addressed molecular storage, because it allows unstable addresses to be identified and considered in future register designs.

Finally, we evaluated the compatibility of the SAMB register output with block-wise parity validation. The three embedded parity bits were used as simple consistency constraints to assess whether the AFM-called register output was compatible with the designed register logic. This analysis was not intended to provide full error correction. Instead, it served as a lightweight validation step for detecting inconsistent blocks and for flagging ambiguous address-level signals as erasures when a confident 0/1 call could not be assigned. Thus, in addition to raw bit-calling accuracy and spatial error mapping, SAMB supports address-level output validation within a two-dimensional molecular coordinate framework.

### 3.5 Erasure statistics and recovery performance

Erasure distributions and parity-based decoding performance were further evaluated using a prespecified independent test set of 56 registers. Fixed height thresholds and decoding rules were applied to the 48 non-FM positions in each register, comprising 45 graph-payload positions and three parity positions.

Among the 2,688 non-FM positions, 462 were classified as erasures, corresponding to an overall erasure rate of 17.19%. These comprised 420 erasures at graph-payload positions ( 420/2,520 , 16.67%) and 42 at parity positions ( 42/168 , 25.00%). Across the 56 registers, $B_{1}$ , $B_{2}$ , and $B_{3}$ contained 101 of 616 (16.40%), 244 of 1,456 (16.76%), and 117 of 616 (18.99%) erased positions, respectively. Thus, the larger number of erasures in $B_{2}$ primarily reflected its larger block size, whereas the position-normalized erasure rate was highest in $B_{3}$ .

The 56 registers comprised 168 parity blocks, of which 18 contained no erasures, 24 contained a single erasure, and 126 contained two or more erasures. Single-erasure decoding was therefore applicable to 24 blocks. Comparison with the designed states showed that 15 of the 24 inferred values were correct (62.50%), whereas nine were incorrect (37.50%). The remaining 438 erasures occurred in blocks containing multiple erasures and were therefore beyond the recovery capacity of a single parity constraint. These findings indicate that successful single-erasure recovery depends not only on correctly identifying the erased position but also on the accuracy of the remaining hard decisions within the same block.

Four readout strategies were subsequently compared using a strict recovery criterion, which required a register to be accepted and all 45 graph-payload bits to match their designed states. Because each register encoded one Petersen-graph payload, strict payload recovery and strict graph recovery were equivalent under this criterion.

The overall strict recovery yields were 1/56 (1.79%) for hard-decision calling, 1/56 (1.79%) for parity-only validation, 0/56 (0.00%) for erasure-only rejection, and 1/56 (1.79%) for erasure-aware parity decoding. Thus, neither parity-only validation nor erasure-aware parity decoding increased the overall strict recovery yield relative to hard-decision calling in this test set, although the hard-decision and erasure-aware approaches recovered different registers. Among accepted outputs, the proportions meeting the strict recovery criterion were 1/56 (1.79%) for hard-decision calling, 1/9 (11.11%) for parity-only validation, not estimable for erasure-only rejection because no register was accepted, and 1/1 (100%) for erasure-aware parity decoding. These results indicate that the observed benefit of parity and erasure processing was improved acceptance selectivity rather than increased overall recovery yield. However, the result for erasure-aware parity decoding was based on only one accepted register and should therefore be interpreted cautiously. Further improvements in signal quality, classification thresholds, and redundancy design may increase the proportion of registers that can be decoded reliably.

## 4 Discussion

In this study, we developed SAMB as a physical two-dimensional DNA molecular register for coordinate-addressed data storage. In contrast to sequence-based DNA storage, where information is encoded along nucleotide order and recovered by population-level sequencing, SAMB stores information as molecular occupancy states at predefined two-dimensional addresses. Its main contribution is therefore not higher storage capacity, but the demonstration that bit information can be placed, read and analyzed within a directly addressable molecular coordinate space. The four-module DNA origami architecture provides the physical basis for this register. Four cross-shaped single modules are assembled into a continuous two-dimensional address space, while the fiducial marker defines the global orientation and coordinate origin. This allows individual SAMB registers to be aligned to the same coordinate frame before decoding. In this design, DNA origami is used not only as a nanoscale scaffold, but also as a molecular register in which bit identity is linked to physical position.

As a proof of concept, we encoded a Petersen graph payload in a 49-coordinate SAMB register. The FM site was used for orientation registration, whereas the remaining 48 non-FM addresses carried 45 graph bits and 3 parity bits. This design converts relational graph information into a two-dimensional molecular occupancy pattern. Using liquid-phase AFM, local height extraction and binary bit calling, the AFM-called register output was used to reconstruct the Petersen graph, linking molecular coordinate readout to graph-level digital information.

A key advantage of coordinate-addressed storage is that readout errors can be traced back to specific molecular addresses. In our population-level analysis, raw bit accuracy, false-positive and false-negative events, and address-level error distributions were quantified across 100 SAMB registers. These results show that SAMB readout can be evaluated statistically, rather than relying only on a single representative molecule. Because each non-FM evaluated position has a known coordinate, uncertain address-level signals can also be considered as erasures in downstream validation. The three parity bits used here serve as simple block-wise consistency checks, rather than a full error-correction scheme. Future designs could build on this coordinate framework by incorporating stronger spatial redundancy and more systematic error-control codes.

The present SAMB system remains a proof-of-concept platform. Its scale is limited by the size and address density of the four-module origami assembly, and AFM-based readout is constrained by imaging throughput, surface preparation and molecular label stability. Further improvements could come from larger or hierarchical DNA origami assemblies, optimized address spacing and molecular labels, and automated image analysis or higher-throughput imaging methods.

Carbon-dot- and other nanomaterial-based systems provide a mechanistically distinct platform for molecular information processing. They generally encode information through changes in fluorescence intensity, emission wavelength, lifetime, or spatial patterns and have been used in concatenated logic libraries, molecular keypad locks, reversible molecular memory, smartphone-readable logic, and anti-counterfeiting applications (Parambil et al., 2023; Vandarkuzhali et al., 2018; Xiao et al., 2021; Kalytchuk et al., 2018). Gold nanoparticle–fluorophore hybrids have also been employed to generate Boolean outputs through plasmonic quenching or fluorescence-lifetime modulation (Barnoy et al., 2019). These platforms offer scalable bulk synthesis, rapid optical readout, tunable chemical responses, and, in some solid-state formulations, good photostability. However, their bit-level scalability and reproducibility may be limited by spectral overlap, signal cross-talk, environmental sensitivity, and batch-to-batch variation. Moreover, many such systems store stimulus-dependent ensemble states rather than independently addressable, high-capacity digital information.

By contrast, the SAMB register encodes binary information through the presence or absence of streptavidin at predefined coordinates on a DNA-origami framework and decodes these states by AFM height measurements. This architecture provides deterministic sequence programmability, nanometre-scale spatial addressability, modular register organization and block-wise parity validation. Its scalability and stability are nevertheless constrained by oligonucleotide cost, multistep assembly, finite origami area, assembly defects, ionic and temperature requirements, nuclease susceptibility, and the relatively low throughput of AFM readout. Thus, nanomaterial platforms are particularly suitable for rapid optical logic, sensing, and security applications, whereas DNA-origami registers are better suited to spatially addressable data representation, error-aware decoding, and programmable nanoscale information processing.

## 5 Conclusion

In this study, we constructed a two-dimensional DNA molecular register based on a spatially addressable molecular breadboard (SAMB). By assembling four cross-shaped DNA origami single modules into a 2×2 framework, SAMB establishes a molecular-scale two-dimensional address space in which each bit is assigned to a predefined coordinate and encoded by the occupancy state of streptavidin at that address. In this way, digital information is organized not as a one-dimensional nucleotide sequence, but as a physical occupancy pattern within a two-dimensional molecular space.

Using a Petersen graph payload in a 49-coordinate register as a proof of concept, we demonstrated a complete workflow from graph-information encoding and coordinate mapping to liquid-phase AFM readout and graph reconstruction. The fiducial marker provides orientation registration and defines the coordinate origin, whereas AFM-resolved height differences enable molecular occupancy states to be converted into a digital register output. Population-level analysis further quantified raw bit accuracy, false-positive and false-negative events, and address-level error distributions, validating the feasibility of coordinate-based readout across multiple SAMB registers.

Overall, SAMB establishes a molecular architecture for physical two-dimensional DNA data storage and address-level readout. This work provides a foundation for developing spatially organized DNA information systems and scalable molecular registers.

## Data Availability

The original contributions presented in the study are included in the article/Supplementary Material, further inquiries can be directed to the corresponding authors.
