# Supplementary material for: Two-dimensional data storage using a DNA molecular register
Source: Front Bioeng Biotechnol. 2026 Sep 11;14:1927944. doi: 10.3389/fbioe.2026.1927944 (PMC13612501; doi:10.3389/fbioe.2026.1927944)
Supplement: Supplementary file 1 [file Supplementaryfile1.docx]

Supplementary Information

**Two-Dimensional Data Storage Using a DNA Molecular Register**

**Supplementary Table 1. Comparison of representative non-sequence nucleic acid memory, rewritable two-dimensional DNA storage, DNA-origami cryptography, and linked DNA-origami storage platforms with SAMB.**

| Study | Architecture | Storage mechanism | Readout method | Orientation registration | Rewritability | Error-control strategy |
| --- | --- | --- | --- | --- | --- | --- |
| Dickinson et al. (2021), dNAM^1^ | Addressable DNA-origami breadboards carrying fountain-coded data droplets. | Presence or absence of DNA-PAINT docking domains represents 1 or 0. | DNA-PAINT super-resolution microscopy. | Dedicated orientation sites enable rotational alignment. | Not shown. | Fountain coding and bi-level parity coding recover lost sites or entire droplets. |
| Pan et al. (2022), 2DDNA^2^ | Oligonucleotide pools encode information in both nucleotide sequences and nick-defined backbone topology. | Sequences store image data; backbone nicks store metadata. | MiSeq sequencing, nick detection, and machine-learning-assisted reconstruction. | Nick-derived reads are aligned to full-length reference sequences; spatial registration is not applicable. | Nick-encoded metadata can be erased by ligation and enzymatically rewritten. | RGB redundancy, discoloration detection, image inpainting, and optional LDPC protection. |
| Zhang et al. (2019), DOC^3^ | DNA origami carrying Braille-like patterns; linked dimers support integrity verification and differential access. | Biotin–streptavidin or fluorescent molecular patterns encode information. | AFM or STORM imaging. | An orientation marker or intrinsic structural asymmetry determines direction. | Not shown. | Majority-based pattern calling and hash verification; no general-purpose ECC. |
| Wisna et al. (2025)^4^ | Two-dimensional rectangular and three-dimensional cuboctahedral DNA origami carrying cryptographic patterns. | Spatial docking-strand patterns are concealed by origami folding and all-DNA steganography. | High-speed DNA-PAINT with clustering and template fitting. | Alignment markers and template-based registration. | Not shown. | Bit redundancy and clustering-assisted classification; accuracy up to approximately 89%, without a complete ECC. |
| Zhang et al. (2025), DONLDS^5^ | Shape-defined DNA-origami nodes with data and pointer domains form linked lists; a 48-bit tetramer was demonstrated. | Streptavidin occupancy encodes bits, while pointer and operation strands control node connections. | Tapping-mode AFM in air. | Orientation/index domains, scaffold loops, and structural asymmetry define reading order. | Node insertion, deletion, connection, and disconnection were demonstrated; individual bits were not rewritten in place. | Cross-verification and threshold-based bit calling; tenfold redundancy with majority voting was modelled, but no ECC was implemented. |
| This work: SAMB | Four cross-shaped modules form a globally registered 49-coordinate physical register. | Forty-five graph bits and three parity bits use streptavidin occupancy; one FM provides registration. | Address-resolved topographic readout by liquid-phase AFM. | A single asymmetric FM defines the global orientation and coordinate origin. | Not shown. | Three block-wise even-parity positions as proof-of-concept design elements and address-level error-frequency analysis; no complete ECC. |

**Note:** “Not shown” indicates that rewriting was not experimentally demonstrated in the corresponding study. SAMB integrates four DNA-origami modules into a single globally registered physical register, allowing direct address-level readout using one common orientation marker.

**Abbreviations:** AFM, atomic force microscopy; ECC, error-correction code; FM, fiducial marker; LDPC, low-density parity-check; STORM, stochastic optical reconstruction microscopy.

**Supplementary Table 2. Oligonucleotide sequences and chemical modifications used in this study.**

| 25[56],27[55] | AGTTTTGCTACGAGGCATAGTAAGAGATGGTT |
| --- | --- |
| 6[55],4[56] | CAGGAAGAACCAGAAGGAAACCGAGAGCAAGA |
| 19[280],21[279] | CCCCTTATCCGGAACCAGAGCCACATGGCTTT |
| 24[71],22[72] | ATTAAGAGTTAATTGCTCCTTTTGTTAGATAC |
| 26[71],24[72] | AAAGGAATCAGAGGGGGTAATAGTTCAAAAAG |
| 10[119],8[120] | GGGTGGTTCCGCTTTCCAGTCGGGCCAGTGCC |
| 22[199],20[200] | GAGGCTGAATTCACAAACAAATAAAGAGCCGC |
| 8[303],9[308] | AGTTGAAAGGAATTGACAGTTGGCAAATCAACGTCTG |
| 1[168],2[152] | TTTTCAAACAAGACAAAGAACGCGATCCGGTA |
| 2[308],3[303] | CTATCTAGATTAAGACGCTGAAAACATAGCGATAGCT |
| 2[183],0[184] | TCCAATCGTATATTTTAGTTAATTGAGCCAGT |
| 0[165],22[200] | ACCGACAAATGAAAGTATTAA |
| 20[103],18[104] | TCATATATTCATTGCCTGAGAGTCAGATTGTA |
| 10[183],8[184] | GAACGAACTCAGTATTAACACCGCACAAACAA |
| 10[303],11[308] | AGAGATAGAACCCTTCGGACATTCTGGCCAACTGCGT |
| 18[103],10[120] | TAAGCAAATCGGATTCTCCGTGTTTTGGAACAAGAGTCCACTGGGCGCCA |
| 3[200],4[184] | GAAACAGTACCTGAGCAAAAGAAGATGATGAA |
| 27[152],26[168] | AAAACGAAAGTTAAAGGCCGCTTTGCTGAGGC |
| 27[216],1[199] | ACGAGGGTAAGCGCGAAACAATTTTAGGCAGAGGCATTTTCTCATCTTC |
| 11[264],10[280] | CAGTCACACGACCAGTAATAAAAGTGACCTGA |
| 26[263],24[264] | CGAGGTGATGCGAATAATAATTTTGAACCCAT |
| 24[135],22[136] | AATCAAAAATGCTGTAGCTCAACAGGAAGTTT |
| 22[39],20[40] | TATTTTCATCTACTAATAGTAGTAAGAAAGGC |
| 23[280],25[279] | AGAACCGCATAGCAAGCCCAATAGTTCACGTT |
| 19[152],18[168] | TAGCATGTAAGGCCGGAAACGTCACACCATTA |
| 4[279],2[280] | ATAACGGACCCTTAGAATCCTTGAGAAGAGTC |
| 1[104],3[103] | TCATCGTACGGGAGGTTTTGAAGCTCAAAAAT |
| 2[151],1[167] | TTCTAAGACAGATATAGAAGGCTTAGAAAACT |
| 7[168],8[152] | CGTTATTACTCGTATTAAATCCTTGTTTTCCC |
| 27[88],28[72] | ATGCGATTTCTTGACAAGAACCGGATATTCAT |
| 6[255],7[231] | TGCACGTAAAAACGGTCAATCATAAGGGAACCCCAGAAGG |
| 11[153],23[119] | AAAGGGCGAATAGCTTAATTGC |
| 24[231],22[232] | AACTACAAGGTGTATCACCGTACTCTATTTCG |
| 20[231],18[232] | GCCACCCTAGTTTGCCTTTAGCGTGGTGAATT |
| 21[56],23[55] | ATCCAATAAATGGTCAATAACCTGGGATTAGA |
| 8[215],6[213] | GAGGATTTCGGAACAAAGAAACCAGAACTGACCAA |
| 17[11],18[16] | AGTGAAACGCCATCAAAAATATTTTTTAACCAATAGG |
| 18[231],17[247] | ATCACCGTAGAAACGCAAAGACACCACGGAAT |
| 6[279],4[280] | AGAACCTACAGGTTTAACGTCAGAGAGAAACA |
| 24[167],25[151] | TCGTCTTTGTTCAGAAAACGAGAATCAAATGC |
| 0[183],25[215] | AATAAGAGAATATAAAGTTTGCCCGGAATACGCCTGTAGCATTCCATTCAACAG |
| 0[308],1[303] | TCACTATATGCGTTATACAAAAAGCCTGTTTAGTATC |
| 28[71],26[72] | TACCCAAAACTTTAATCATTGTGATAACGCCA |
| 25[216],27[215] | TTTCAGCGCCGACAATGACAACAAGCATCGGA |
| 0[279],1[263] | AGTATAAAGCCAACGCTCAACAGTAGAATAAA |
| 11[232],10[248] | CTGAAATGGATTATTTACATTGGCCAATATTT |
| 20[263],18[264] | CCGCCTCCTTCATCGGCATTTTCGAAGGTAAA |
| 20[199],0[120] | CACCAGAATAGCAGCACCGTAATCTTGGGAATTTACCGCGCAATAAACA |
| 19[88],21[87] | CTATCAGGTTTAAATGCAATGCCTCAAAGAAT |
| 7[40],9[39] | CCATTCGCGCTGTTTCCTGTGTGAACGAGCCG |
| 26[199],24[200] | CACGCATAATTTTGCTAAACAACTCAGACAGC |
| 28[143],26[136] | CGGTGTACAGACCAGGTTGGGAAGAAAAATCTATTACAGG |
| 17[280],19[279] | ATATGGTTACCGATTGAGGGAGGGGTCATAGC |
| 1[136],3[135] | AAGCAAATACGCGAGGCGTTTTAGAATAACAT |
| 4[87],2[88] | GAATTGAGGATTTTTTGTTTAACGCTTAAATC |
| 22[308],21[308] | GCACGATAAGTTTTAACGGGGCAGGAGTGTACTGGTAGCACG |
| 5[264],7[263] | TAGATTTTCCATATCAAAATTATTATTATCAG |
| 27[280],28[264] | TAAACGGGCCTAAAACGAAAGAGGCAAAAGAA |
| 3[168],4[152] | TAACAATTCAAGAAAACAAAATTAGGAGAATT |
| 5[235],4[248] | CGAGGCGCAGCAGAAATAAAGGAATACCA |
| 9[232],11[231] | ATCTAAAGCTATTAGTCTTTAATGTCAATCGT |
| 6[308],7[308] | CAGACATACTTCTGAATAATGCCTGATTGTTTGGATTCAGAC |
| 11[40],10[56] | GTTCCGAAATCGGCAAAATCCCTTTGGCCCTG |
| 25[248],27[247] | AAAGGAATATTTCTTAAACAGCTTGCTTTGAG |
| 11[11],10[16] | ATGTAAGGCGAAAATCCTGTTGCTGGTTTGCCCCAGC |
| 28[308],27[303] | TACATACTACGAAGGCACCAATAAAATACGTAATGCC |
| 9[11],8[16] | GCTCGCAATTCCACACAACATAATTGTTATCCGCTCA |
| 24[39],22[40] | GCGTTTTAAAACTCCAACAGGTCATTTAGCTA |
| 1[264],3[263] | CACCGGAAATTTATCAAAATCATAGCTATTAA |
| 20[167],21[151] | TTGACAGGATACTTTTGCGGGAGAACATTATG |
| 27[16],28[11] | GAAACACCAGAACGAGAGGCTTGCCCTGACGAACGCA |
| 4[308],5[308] | ACGCAGTACCTTTTACATCGGTGAATATACAGTAACAACGCA |
| 9[200],28[104] | GTGCCACGTAAAACATTTCAGGACGCGCATAGGCTGGCTGACCTTCATC |
| 0[55],1[39] | TAATATCCCATCCTAATTTACGAGTCCTTATC |
| 23[11],24[11] | GTCTGGAACCAGACCGGAAGCATTCGAGCTTCAAAGCGTCTG |
| 7[232],9[231] | AGCGGAATGAGCCGTCAATAGATAAATGAAAA |
| 7[200],9[199] | TCATTTTGAGAAGTATTAGACTTTCTGCAACA |
| 3[40],5[39] | ATAAACAGAATAGCAATAGCTATCATAGCCGA |
| 5[75],7[71] | AATAAAGGGGACGACGACAGTCTGTTGGG |
| 25[280],27[279] | GAAAATCTTGTATCGGTTTATCAGGTTTCCAT |
| 18[71],17[87] | TTTGTTAAATCAACATTAAATGTGAGCGAGTA |
| 3[72],5[74] | TAAGAAACTTAAGCCCAATAATAAGGAAACGCAAT |
| 23[184],2[184] | AGTGCCGTTTAGCGTAACGATCTAGAATTTTCTATTACCTTGATGCAAA |
| 17[176],19[183] | ATGTTAGCAAACGTAGGCAAAATCACCAGTAGCCAATGAA |
| 21[280],23[279] | TGATGATATCAGTGCCTTGAGTAACCACCCTC |
| 21[248],23[245] | TTACCGTTAGTTAATGCCCCCTGCCAGGAG |
| 23[160],22[168] | GTTTTGCTCAGTACCAGATTAGGA |
| 0[87],1[71] | ATCAACAATAGATAAGTCCTGAACAGTACCGC |
| 18[39],17[55] | TCAGCTCAATTCGCGTCTGGCCTTCCTGTAGC |
| 10[279],8[280] | AAGCGTAATCAATCAATATCTGGTGGAAGGTT |
| 11[200],10[216] | ATGGAAATACCTACATTTTGACGCCGCGAACT |
| 0[145],1[135] | TTCTGTCCAGACGACGACCCAATAGC |
| 10[55],8[56] | AGAGAGTTAAGTGTAAAGCCTGGGCGTAATCA |
| 24[103],10[184] | TAGTCAGAGCGGATGGCTTAGTAAACCGTCTATCAGCCATTGCAACAGGAAAAAATACC |
| 3[16],2[11] | AGAGCCTAATTTGCCATAACGAGCGTCTTTCCGCACG |
| 2[215],28[200] | GTTATATAATTTAATGGTTTGAAACGCCAACATGTAATAGTACAACGGA |
| 22[231],20[232] | GAACCTATGAATGGAAAGCGCAGTTCAGAACC |
| 21[88],23[85] | TAGCAAAAAGATTTAGTTTGACCAATAAGA |
| 9[104],17[119] | CTCACTGCTTTCTTTTCACCAGTGAGTGTTGTTCCAGTGGAACAAACG |
| 18[135],17[175] | ATCAGAAAACCGTAATGGGATAGGTCACGTTGGTGTAGATCTCCTTATTACGCAGT |
| 4[119],2[120] | TTGAGCGCCAGCCTTTACAGAGAGCGAACCTC |
| 0[119],1[103] | ACATGTTCAGCTAATGCAGAACGCTTTTATTT |
| 10[87],8[88] | AACAGCTGCTAACTCACATTAATTTCTAGAGG |
| 10[215],8[216] | GATAGCCCCTGAGAGCCAGCAGCAATACATTT |
| 9[136],11[135] | CGTGCCAGAGGCGGTTTGCGTATTATTAAAGA |
| 22[167],23[159] | TTAGCGGGCTAAAGTACGGTGTCTTGTTTTAAATATGCAA |
| 8[247],7[255] | ATAGATTATATCATCATATTCCTG |
| 4[151],3[167] | AACTGAACGGAAGCGCATTAGACGATTACATT |
| 11[72],10[88] | AAAGAATAGCCCGAGATAGGGTTGAGACGGGC |
| 10[151],9[167] | GCGGGGAGCTGCATTAATGAATCGAACAGAGG |
| 8[183],7[199] | TTCGACAAATTTTAAAAGTTTGAGTAACATTA |
| 17[216],19[215] | CATATAAACACCGACTTGAGCCATAGTAGCGA |
| 28[103],26[104] | AAGAGTAATTAAGAACTGGCTCATTAGGAATA |
| 0[247],1[231] | ATTGAGAATCGCCATATTTAACAATACCGACC |
| 19[120],21[119] | ACAAGAGACAAGGATAAAAATTTTCAGAGCAT |
| 4[247],2[248] | AGTTACAAGCTTCTGTAAATCGTCGGTCTGAG |
| 18[263],17[279] | TATTGACGTTTGTCACAATCAATAGAAAATTC |
| 22[263],20[264] | GTATAAACCCAGTAAGCGTCATACCACCGGAA |
| 7[104],9[103] | CGCCAGCTATGCCTGCAGGTCGACGCGTTGCG |
| 1[16],0[11] | AATAATCGGCTGTCTTCATGTAGAAACCAATCGTATG |
| 27[56],28[40] | TAATTTCATCAACGTAACAAAGCTGCTCATTC |
| 26[135],24[136] | TAGAAAGAATTCATTGAATCCCCCTGACCATA |
| 19[56],21[55] | GAGGGTAGAAGATTCAAAAGGGTGGCATTAAC |
| 5[11],4[11] | CGTGCTAAGAAAAGTAAGCAGTTACCGAAGCCCTTTTCGTGC |
| 8[87],6[88] | ATCCCCGGTCGGTGCGGGCCTCTTTGCATCTG |
| 2[247],0[248] | AGACTACCAATAAGGCGTTAAATAAGGGCTTA |
| 9[264],11[263] | TCAAACCCGAATACGTGGCACAGAAGATTCAC |
| 2[87],0[88] | AAGATTAGAGAACAAGCAAGCCGTGCCTGTTT |
| 8[55],6[56] | TGGTCATACATTCAGGCTGCGCAAATCGGCCT |
| 8[151],7[167] | AGTCACGAGTTGGGTAACGCCAGGTGCCCGAA |
| 20[71],18[72] | TGTAGGTACTATTTTTGAGAGATCGTTAATAT |
| 19[11],20[16] | GATAGTATGATATTCAACCGTGTCAAATCACCATCAA |
| 28[263],26[264] | TACACTAAACTTTTTCATGAGGAACTTGCTTT |
| 23[120],25[119] | TGAATATAATCAGGTCTTTACCCTCGGAATCG |
| 21[152],20[168] | ACCCTGTAAGGTTGAGGCAGGTCACGCCAGCA |
| 24[308],23[308] | ATCTAACCCTCATTTTCAGGGCACCCTCAGAGCCACCATCTA |
| 19[216],21[215] | CAGAATCACAGAGCCACCACCCTCATCCTCAT |
| 9[40],11[39] | GAAGCATAGCAGCAAGCGGTCCACTGATGGTG |
| 8[119],7[135] | AAGCTTGCGGCGAAAGGGGGATGTGCTGCAAG |
| 7[72],9[71] | AAGGGCGAGTACCGAGCTCGAATTGTGCCTAA |
| 23[246],25[247] | GTTTAGTACCACACTGAGTTTCGTCAGAACAACT |
| 24[263],22[264] | GTACCGTAGCCACCCTCAGAACCGCAGTGCCC |
| 4[215],2[216] | TTTCAATTACATAAATCAATATATTAGGTTGG |
| 28[199],26[200] | GATTTGTATCAGCAGCGAAAGACACCATCGCC |
| 6[107],7[103] | GCATCGTAACCGCGCTATTA |
| 1[200],3[199] | TGACCTAAACTATATGTAAATGCTTTTTAATG |
| 21[216],0[146] | TAAAGCCATATTCTGAAACATTAGGTAAAGTAA |
| 21[120],22[106] | AAAGCTAAATAACAGTTGATTCCCAATTCT |
| 8[279],6[280] | ATCTAAAAAATTCATCAATATAATGAAGGGTT |
| 20[303],19[308] | TTCATAATCAAAATCATAGCGTTTGCCATCTTCGTGC |
| 6[87],4[88] | CCAGTTTGCGGAATACCCAAAAGAACCCACAA |
| 28[39],26[40] | AGTGAATATAGTAAATTGGGCTTGAGCAACAC |
| 19[248],21[247] | AGCGCGTTCTCAGAGCCGCCACCCCTCTGAAT |
| 3[232],5[234] | ATAACCTTAATCGCGCAGAGGCGACTTAGCCGGAA |
| 25[120],11[199] | TCATAAATTTCATCAGTTGAGATTTATACCAGTCGCCATTAAAACGCTC |
| 26[167],27[151] | TTGCAGGGCTAACGGAACAACATTACGTTAAT |
| 18[167],19[151] | CCATTAGCCAATCATATGTACCCCCGTAAAAC |
| 23[56],25[55] | GAGTACCTGAAGCCCGAAAGACTTGCAAAAGA |
| 1[72],3[71] | ACTCATCGTTGCTATTTTGCACCCAATCCAAA |
| 25[88],27[87] | TAGACTGGAACTAATGCAGATACAATTACCTT |
| 3[104],5[106] | GAAAATAGTAATATCAGAGAGATAACTGGCATGAT |
| 26[103],24[104] | CCACATTCATAGCGTCCAATACTGGACTATTA |
| 26[308],25[303] | CGAGCAAAAGGAGCCTTTAATCCAAAAAAAAGGCTCC |
| 21[184],23[183] | GCCTTGATGACTCCTCAAGAGAAGGGCGGATA |
| 25[16],26[11] | GACGATAAAAACCAAACCCTCGTTTACCAGACCAGAC |
| 21[11],22[11] | TGCGTAAAAGGTGGCATCAATTTTGGGGCGCGAGCTGTGCGT |
| 28[231],26[232] | ATTATACCAGCAACGGCTACAGAGGATACCGA |
| 27[248],28[232] | GACTAAAGAACACTCATCTTTGACCCCCAGCG |
| 3[264],5[263] | TTAATTTTTTCGCCTGATTGCTTTAAATTGCG |
| 10[247],8[248] | TTGAATGGCATCACCTTGCTGAACAACAACTA |
| 22[71],20[72] | ATTTCGCAAATCATACAGGCAAGGGAGTAATG |
| 7[11],6[11] | TAGATTGGTGCCGGAAACCAGTTTCCGGCACCGCTTCTAGAT |
| 17[248],19[247] | AAGTTTATGAAATTATTCATTAAACAGACTGT |
| 19[184],21[183] | ACCATCGACCACCACCAGAGCCGCGACGATTG |
| 2[55],0[56] | TTTTATCCAACGGGTATTAAACCAAAGAAAAA |
| 1[40],3[39] | ATTCCAAGTGAATCTTACCAACGCGTTACAAA |
| 22[135],9[135] | CATTCCATATCGGTTGTACCAAAAAGCCTTTATAACGACGGAAACCTGT |
| 26[231],24[232] | TAGTTGCGGAGTGAGAATAGAAAGCCAGTACA |
| 7[136],18[136] | GCGATTAACGTTGTAATTTTCAACGATCGATGAACGGTAATGGTTGATA |
| 17[88],19[87] | ACAACCCGTATTTAAATTGTAAACTACAAAGG |
| 5[40],7[39] | ACAAAGTTTCGCACTCCAGCCAGCGCAAAGCG |
| 24[199],23[213] | CCTCATAGCGAGAGGGTTGATATAAGTATA |
| 17[120],19[119] | GCGGATTGAGCCCCAAAAACAGGATGGAGCAA |
| 9[72],11[71] | TGAGTGAGATTGCCCTTCACCGCCATAAATCA |
| 17[56],19[55] | CAGCTTTCAATTCGCATTAAATTTATGCCGGA |
| 7[264],9[263] | ATGATGGCTATCTTTAGGAGCACTCTCAAATA |
| 25[152],24[168] | TTTAAACACCAGACGTTAGTAAATAAGTTTTG |
| 27[184],28[144] | CGTCACCCTCATCGCCTGATAAATTGTGTCGAAATCCGCGAAAGAGGACAGATGAA |
| 20[39],18[40] | CGGAGACATCTAGCTGATAAATTATTGTTAAA |
| 1[232],3[231] | GTGTGATATTTTTAACCTCCGGCTGTGAGTGA |
| 23[86],25[87] | GGTCATTTTTAGCAAAGCGGATTGCAAAAATGTT |
| 3[136],4[120] | AAAAACAGACCCTGAACAAAGTCAGAGGGTAA |
| 4[55],2[56] | AACAATGACCATATTATTTATCCCAGCTACAA |
| 18[303],17[308] | AAAAGGGCGACATTCATACCAGCGCCAAAGACCATAC |
| 9[168],10[152] | TGAGGCGGCACCAGCAGAAGATAAGCCAACGC |
| 4[183],27[183] | ACAAACATTCATTTGATTGTATGGGACCGATATATTCGGTCTGCGGGAT |
| 26[39],24[40] | TATCATAAATAGCGAGAGGCTTTTCAAATATC |
| 11[136],20[104] | ACGTGGACTCCAACGTCTTGCGAACGAGTTTAAGCAATAAAGCCTTAGAACCC |
| 2[279],0[280] | AATAGTGATCATAATTACTAGAAATTCTTACC |
| 2[119],17[215] | CCGACTTGGGAATCATTTAGAGCCAAAAATACATACATAAAGGTGGCAA |
| 5[212],4[216] | TGCTCCATGTTAATTATTCA |
| 17[11],18[16]D-L | ACTCGAACGCCATCAAAAATATTTTTTAACCAATAGG |
| 11[11],10[16]D-L | CGAGTAGGCGAAAATCCTGTTGCTGGTTTGCCCCAGC |
| 19[11],20[16]D-L | AATCTTATGATATTCAACCGTGTCAAATCACCATCAA |
| 9[11],8[16]D-L | AGATTCAATTCCACACAACATAATTGTTATCCGCTCA |
| 21[11],22[11]D-L | GCACGAAAAGGTGGCATCAATTTTGGGGCGCGAGCTGCGAAA |
| 7[11],6[11]D-L | CGTGCTGGTGCCGGAAACCAGTTTCCGGCACCGCTTCTTTCG |
| 23[11],24[11]D-L | AAGAGGAACCAGACCGGAAGCATTCGAGCTTCAAAGCGACTT |
| 5[11],4[11]D-L | CTCTTTAAGAAAAGTAAGCAGTTACCGAAGCCCTTTTAAGTC |
| 25[16],26[11]D-L | GACGATAAAAACCAAACCCTCGTTTACCAGACGAGAC |
| 3[16],2[11]D-L | AGAGCCTAATTTGCCATAACGAGCGTCTTTCCGTCTC |
| 27[16],28[11]D-L | GAAACACCAGAACGAGAGGCTTGCCCTGACGATATAG |
| 1[16],0[11]D-L | AATAATCGGCTGTCTTCATGTAGAAACCAATCCTATA |
| 18[303],17[308]U-R | AAAAGGGCGACATTCATACCAGCGCCAAAGACAATGC |
| 10[303],11[308]U-R | AGAGATAGAACCCTTCGGACATTCTGGCCAACGCATT |
| 20[303],19[308]U-R | TTCATAATCAAAATCATAGCGTTTGCCATCTTGTCAA |
| 8[303],9[308]U-R | AGTTGAAAGGAATTGACAGTTGGCAAATCAACTTGAC |
| 22[308],21[308]U-R | GCCAGATAAGTTTTAACGGGGCAGGAGTGTACTGGTATCGAC |
| 6[308],7[308]U-R | CTGGCATACTTCTGAATAATGCCTGATTGTTTGGATTGTCGA |
| 24[308],23[308]U-R | GTTACACCCTCATTTTCAGGGCACCCTCAGAGCCACCGCCTA |
| 4[308],5[308]U-R | GTAACGTACCTTTTACATCGGTGAATATACAGTAACATAGGC |
| 26[308],25[303]U-R | GAGCTAAAAGGAGCCTTTAATCCAAAAAAAAGGCTCC |
| 2[308],3[303]U-R | AGCTCTAGATTAAGACGCTGAAAACATAGCGATAGCT |
| 28[308],27[303]U-R | TACGAACTACGAAGGCACCAATAAAATACGTAATGCC |
| 0[308],1[303]U-R | TCGTAATATGCGTTATACAAAAAGCCTGTTTAGTATC |

| 2[55]0[56]-L1bio | TTTTATCCAACGGGTATTAAACCAAAGTTT |
| --- | --- |
| 2[87]0[88]-L2bio | AAGATTAGAGAACAAGCAAGCCGTGCCTTT |
| 9[40]11[39]-L3bio | GAAGCATAGCAGCAAGCGGTCCACTGATGGTGTTT |
| 9[72]11[71]-L4bio | TGAGTGAGATTGCCCTTCACCGCCATAAATCATTT |
| 2[247]0[248]R1bio | AGACTACCAATAAGGCGTTAAATAAGGTTT |
| 2[279]0[280]R2bio | AATAGTGATCATAATTACTAGAAATTCTTT |
| 9[232]11[231]R3bio | ATCTAAAGCTATTAGTCTTTAATGTCAATCGTTTT |
| 9[264]11[263]R4bio | TCAAACCCGAATACGTGGCACAGAAGATTCACTTT |
| 18[39]17[55]-D1bio | TCAGCTCAATTCGCGTCTGGCCTTCCTGTAGCTTT |
| 18[71]17[87]-D2bio | TTTGTTAAATCAACATTAAATGTGAGCGAGTATTT |
| 27[56]28[40]-D3bio | TAATTTCATCAACGTAACAAAGCTGCTTTT |
| 27[88]28[72]-D4bio | ATGCGATTTCTTGACAAGAACCGGATATTT |
| 18[231]17[247]U1bio | ATCACCGTAGAAACGCAAAGACACCACGGAATTTT |
| 18[263]17[279]U2bio | TATTGACGTTTGTCACAATCAATAGAAAATTCTTT |
| 27[248]28[232]U3bio | GACTAAAGAACACTCATCTTTGACCCCTTT |
| 27[280]28[264]U4bio | TAAACGGGCCTAAAACGAAAGAGGCAATTT |

| L1bio-SD | AAACTTTGGTTTAATACCCGTTGGATAAAA |
| --- | --- |
| L2bio-SD | AAAGGCACGGCTTGCTTGTTCTCTAATCTT |
| L3bio-SD | AAACACCATCAGTGGACCGCTTGCTGCTATGCTTC |
| L4bio-SD | AAATGATTTATGGCGGTGAAGGGCAATCTCACTCA |
| R1bio-SD | AAACCTTATTTAACGCCTTATTGGTAGTCT |
| R2bio-SD | AAAGAATTTCTAGTAATTATGATCACTATT |
| R3bio-SD | AAAACGATTGACATTAAAGACTAATAGCTTTAGAT |
| R4bio-SD | AAAGTGAATCTTCTGTGCCACGTATTCGGGTTTGA |
| D1bio-SD | AAAGCTACAGGAAGGCCAGACGCGAATTGAGCTGA |
| D2bio-SD | AAATACTCGCTCACATTTAATGTTGATTTAACAAA |
| D3bio-SD | AAAAGCAGCTTTGTTACGTTGATGAAATTA |
| D4bio-SD | AAATATCCGGTTCTTGTCAAGAAATCGCAT |
| U1bio-SD | AAAATTCCGTGGTGTCTTTGCGTTTCTACGGTGAT |
| U2bio-SD | AAAGAATTTTCTATTGATTGTGACAAACGTCAATA |
| U3bio-SD | AAAGGGGTCAAAGATGAGTGTTCTTTAGTC |
| U4bio-SD | AAATTGCCTCTTTCGTTTTAGGCCCGTTTA |

| FM-bio | TAAGAAACTTAAGCCCAATAATAAGGAAACGCAATTTTCCTCTAG |
| --- | --- |

**Supplementary Fig. 1**

**Supplementary Fig. 1 | Full coordinate map of the 49-coordinate SAMB register.** Complete coordinate layout of the SAMB molecular register. The fiducial marker (FM) defines the coordinate origin (0,0) and global orientation. The remaining non-FM coordinate-defined addresses are distributed across four single modules (SM1–SM4). Yellow sites indicate logic 1 addresses with SA present, grey sites indicate logic 0 addresses without SA, and purple sites indicate parity-bit positions.

**Supplementary Table 3. Coordinate mapping of non-FM molecular addresses across SM1–SM4.**

| SM1 | SM2 | SM3 | SM4 |
| --- | --- | --- | --- |
| (-3,3) | (2,3) | (-3,-2) | (2,-2) |
| (-2,3) | (3,3) | (-2,-2) | (3,-2) |
| (-2,2) | (3,2) | (-2,-3) | (3,-3) |
| (-3,2) | (2,2) | (-3,-3) | (2,-3) |
| (-3,4) | (2,4) | (-3,-1) | (2,-1) |
| (-2,4) | (3,4) | (-2,-1) | (3,-1) |
| (-1,3) | (4,3) | (-1,-2) | (4,-2) |
| (-1,2) | (4,2) | (-1,-3) | (4,-3) |
| (-2,1) | (3,1) | (-1,-4) | (3,-4) |
| (-3,1) | (2,1) | (-3,-4) | (2,-4) |
| (-4,2) | (1,2) | (-4,-3) | (1,-3) |
| (-4,3) | (1,3) | (-4,-2) | (1,-2) |

**Supplementary Table 4. Assignment of FM, graph-bit and parity-bit positions in the 49-coordinate SAMB register.**

| **Register position / Bit index** | $\boldsymbol{P}_{\boldsymbol{i}}\boldsymbol{(x,y)}$ | **SM** | **Type** | **Designed value** | **Molecular state** | **Graph meaning** |
| --- | --- | --- | --- | --- | --- | --- |
| FM | $P_{0}$(0,0) | SM1 | fiducial marker | 1 | SA present | orientation marker |
| 1 | $P_{1}$(-3,3) | SM1 | graph bit | 1 | SA present | A1,2 adjacency between nodes 1 and 2 |
| 2 | $P_{2}$ (-2,3) | SM1 | graph bit | 0 | SA absent | A1,3 adjacency between nodes 1 and 3 |
| 3 | $P_{3}$ (-2,2) | SM1 | graph bit | 0 | SA absent | A1,4 adjacency between nodes 1 and 4 |
| 4 | $P_{4}$ (-3,2) | SM1 | graph bit | 1 | SA present | A1,5 adjacency between nodes 1 and 5 |
| 5 | $P_{5}$ (-3,4) | SM1 | graph bit | 1 | SA present | A2,3 adjacency between nodes 2 and 3 |
| 6 | $P_{6}$ (-2,4) | SM1 | graph bit | 0 | SA absent | A2,4 adjacency between nodes 2 and 4 |
| 7 | $P_{7}$ (-1,3) | SM1 | graph bit | 0 | SA absent | A2,5 adjacency between nodes 2 and 5 |
| 8 | $P_{8}$ (-1,2) | SM1 | graph bit | 1 | SA present | A3,4 adjacency between nodes 3 and 4 |
| 9 | $P_{9}$ (-2,1) | SM1 | graph bit | 0 | SA absent | A3,5 adjacency between nodes 3 and 5 |
| 10 | $P_{10}$ (-3,1) | SM1 | graph bit | 1 | SA present | A4,5 adjacency between nodes 4 and 5 |
| 11 | $P_{11}$ (-4,2) | SM1 | graph bit | 1 | SA present | A5,10 bridging edge between nodes 5 and 10 |
| 12 | $P_{12}$ (-4,3) | SM1 | parity bit | 1 | SA present | Parity check for section I |
| 13 | $P_{13}$ (2,3) | SM2 | graph bit | 1 | SA present | A1,6 bridging edge between nodes 1 and 6 |
| 14 | $P_{14}$ (3,3) | SM2 | graph bit | 0 | SA absent | A1,7 bridging edge between nodes 1 and 7 |
| 15 | $P_{15}$ (3,2) | SM2 | graph bit | 0 | SA absent | A1,8 bridging edge between nodes 1 and 8 |
| 16 | $P_{16}$ (2,2) | SM2 | graph bit | 0 | SA absent | A1,9 bridging edge between nodes 1 and 9 |
| 17 | $P_{17}$ (2,4) | SM2 | graph bit | 0 | SA absent | A1,10 bridging edge between nodes 1 and 10 |
| 18 | $P_{18}$ (3,4) | SM2 | graph bit | 0 | SA absent | A2,6 bridging edge between nodes 2 and 6 |
| 19 | $P_{19}$ (4,3) | SM2 | graph bit | 1 | SA present | A2,7 bridging edge between nodes 2 and 7 |
| 20 | $P_{20}$ (4,2) | SM2 | graph bit | 0 | SA absent | A2,8 bridging edge between nodes 2 and 8 |
| 21 | $P_{21}$ (3,1) | SM2 | graph bit | 0 | SA absent | A2,9 bridging edge between nodes 2 and 9 |
| 22 | $P_{22}$ (2,1) | SM2 | graph bit | 0 | SA absent | A2,10 bridging edge between nodes 2 and 10 |
| 23 | $P_{23}$ (1,2) | SM2 | graph bit | 0 | SA absent | A3,6 bridging edge between nodes 3 and 6 |
| 24 | $P_{24}$ (1,3) | SM2 | graph bit | 0 | SA absent | A3,7 bridging edge between nodes 3 and 7 |
| 25 | $P_{25}$ (-3,-2) | SM3 | graph bit | 1 | SA present | A3,8 bridging edge between nodes 3 and 8 |
| 26 | $P_{26}$ (-2,-2) | SM3 | graph bit | 0 | SA absent | A3,9 bridging edge between nodes 3 and 9 |
| 27 | $P_{27}$ (-2,-3) | SM3 | graph bit | 0 | SA absent | A3,10 bridging edge between nodes 3 and 10 |
| 28 | $P_{28}$ (-3,-3) | SM3 | graph bit | 0 | SA absent | A4,6 bridging edge between nodes 4 and 6 |
| 29 | $P_{29}$ (-3,-1) | SM3 | graph bit | 0 | SA absent | A4,7 bridging edge between nodes 4 and 7 |
| 30 | $P_{30}$ (-2,-1) | SM3 | graph bit | 0 | SA absent | A4,8 bridging edge between nodes 4 and 8 |
| 31 | $P_{31}$ (-1,-2) | SM3 | graph bit | 1 | SA present | A4,9 bridging edge between nodes 4 and 9 |
| 32 | $P_{32}$ (-1,-3) | SM3 | graph bit | 0 | SA absent | A4,10 bridging edge between nodes 4 and 10 |
| 33 | $P_{33}$ (-1,-4) | SM3 | graph bit | 0 | SA absent | A5,6 bridging edge between nodes 5 and 6 |
| 34 | $P_{34}$ (-3,-4) | SM3 | graph bit | 0 | SA absent | A5,7 bridging edge between nodes 5 and 7 |
| 35 | $P_{35}$ (-4,-3) | SM3 | graph bit | 0 | SA absent | A5,8 bridging edge between nodes 5 and 8 |
| 36 | $P_{36}$ (-4,-2) | SM3 | graph bit | 0 | SA absent | A5,9 bridging edge between nodes 5 and 9 |
| 37 | $P_{37}$ (2,-2) | SM4 | graph bit | 0 | SA absent | A6,7 adjacency between nodes 6 and 7 |
| 38 | $P_{38}$ (3,-2) | SM4 | graph bit | 1 | SA present | A6,8 adjacency between nodes 6 and 8 |
| 39 | $P_{39}$ (3,-3) | SM4 | graph bit | 1 | SA present | A6,9 adjacency between nodes 6 and 9 |
| 40 | $P_{40}$ (2,-3) | SM4 | graph bit | 0 | SA absent | A6,10 adjacency between nodes 6 and 10 |
| 41 | $P_{41}$ (2,-1) | SM4 | graph bit | 0 | SA absent | A7,8 adjacency between nodes 7 and 8 |
| 42 | $P_{42}$ (3,-1) | SM4 | graph bit | 1 | SA present | A7,9 adjacency between nodes 7 and 9 |
| 43 | $P_{43}$ (4,-2) | SM4 | graph bit | 1 | SA present | A7,10 adjacency between nodes 7 and 10 |
| 44 | $P_{44}$ (4,-3) | SM4 | graph bit | 0 | SA absent | A8,9 adjacency between nodes 8 and 9 |
| 45 | $P_{45}$ (3,-4) | SM4 | graph bit | 1 | SA present | A8,10 adjacency between nodes 8 and 10 |
| 46 | $P_{46}$ (2,-4) | SM4 | graph bit | 0 | SA absent | A9,10 adjacency between nodes 9 and 10 |
| 47 | $P_{47}$ (1,-3) | SM4 | parity bit | 1 | SA present | Parity check for section II |
| 48 | $P_{48}$ (1,-2) | SM4 | parity bit | 1 | SA present | Parity check for section III |

**Supplementary Fig. 2**

**Supplementary Fig. 2 | Fiducial-marker-dependent orientation registration of the SAMB register.** (a) Representative AFM image of a SAMB register without a fiducial marker (FM), showing the symmetric four-module architecture with ambiguous rotational orientation. (b) Representative AFM image of a SAMB register with an FM. The asymmetric FM provides a positional reference for defining the global orientation and coordinate registration of the SAMB register. Scale bars, 100 nm.

**Supplementary Fig. 3**

**Supplementary Fig. 3 | Height-threshold calibration for bit calling using 300 molecular address sites.** (a) Height distributions of designed 0 and designed 1 address sites measured from 300 predefined molecular addresses. Each point represents one address-site measurement, and boxes indicate the median and interquartile range. The dashed line marks the 3.7 nm threshold used for binary bit calling. (b) Height-frequency distributions of designed 0 and designed 1 address populations. The vertical dashed line indicates the selected 3.7 nm threshold.

**Supplementary Table 5 | Per-register raw bit accuracy calculated from 48 non-FM evaluated positions.**

| **Register ID** | **Non-FM positions analyzed** | **Correct raw bit calls** | **Incorrect raw bit calls** | **Raw bit accuracy (%)** |
| --- | --- | --- | --- | --- |
| SAMB-001 | 48 | 44 | 4 | 91.667 |
| SAMB-002 | 48 | 44 | 4 | 91.667 |
| SAMB-003 | 48 | 37 | 11 | 77.083 |
| SAMB-004 | 48 | 45 | 3 | 93.750 |
| SAMB-005 | 48 | 38 | 10 | 79.167 |
| SAMB-006 | 48 | 37 | 11 | 77.083 |
| SAMB-007 | 48 | 40 | 8 | 83.333 |
| SAMB-008 | 48 | 41 | 7 | 85.417 |
| SAMB-009 | 48 | 39 | 9 | 81.250 |
| SAMB-010 | 48 | 42 | 6 | 87.500 |
| SAMB-011 | 48 | 40 | 8 | 83.333 |
| SAMB-012 | 48 | 41 | 7 | 85.417 |
| SAMB-013 | 48 | 45 | 3 | 93.750 |
| SAMB-014 | 48 | 38 | 10 | 79.167 |
| SAMB-015 | 48 | 37 | 11 | 77.083 |
| SAMB-016 | 48 | 41 | 7 | 85.417 |
| SAMB-017 | 48 | 36 | 12 | 75.000 |
| SAMB-018 | 48 | 42 | 6 | 87.500 |
| SAMB-019 | 48 | 38 | 10 | 79.167 |
| SAMB-020 | 48 | 44 | 4 | 91.667 |
| SAMB-021 | 48 | 39 | 9 | 81.250 |
| SAMB-022 | 48 | 42 | 6 | 87.500 |
| SAMB-023 | 48 | 41 | 7 | 85.417 |
| SAMB-024 | 48 | 38 | 10 | 79.167 |
| SAMB-025 | 48 | 38 | 10 | 79.167 |
| SAMB-026 | 48 | 41 | 7 | 85.417 |
| SAMB-027 | 48 | 39 | 9 | 81.250 |
| SAMB-028 | 48 | 33 | 15 | 68.750 |
| SAMB-029 | 48 | 48 | 0 | 100.000 |
| SAMB-030 | 48 | 39 | 9 | 81.250 |
| SAMB-031 | 48 | 41 | 7 | 85.417 |
| SAMB-032 | 48 | 39 | 9 | 81.250 |
| SAMB-033 | 48 | 45 | 3 | 93.750 |
| SAMB-034 | 48 | 45 | 3 | 93.750 |
| SAMB-035 | 48 | 46 | 2 | 95.833 |
| SAMB-036 | 48 | 43 | 5 | 89.583 |
| SAMB-037 | 48 | 45 | 3 | 93.750 |
| SAMB-038 | 48 | 44 | 4 | 91.667 |
| SAMB-039 | 48 | 45 | 3 | 93.750 |
| SAMB-040 | 48 | 37 | 11 | 77.083 |
| SAMB-041 | 48 | 43 | 5 | 89.583 |
| SAMB-042 | 48 | 43 | 5 | 89.583 |
| SAMB-043 | 48 | 45 | 3 | 93.750 |
| SAMB-044 | 48 | 44 | 4 | 91.667 |
| SAMB-045 | 48 | 45 | 3 | 93.750 |
| SAMB-046 | 48 | 38 | 10 | 79.167 |
| SAMB-047 | 48 | 48 | 0 | 100.000 |
| SAMB-048 | 48 | 43 | 5 | 89.583 |
| SAMB-049 | 48 | 48 | 0 | 100.000 |
| SAMB-050 | 48 | 37 | 11 | 77.083 |
| SAMB-051 | 48 | 33 | 15 | 68.750 |
| SAMB-052 | 48 | 45 | 3 | 93.750 |
| SAMB-053 | 48 | 46 | 2 | 95.833 |
| SAMB-054 | 48 | 44 | 4 | 91.667 |
| SAMB-055 | 48 | 44 | 4 | 91.667 |
| SAMB-056 | 48 | 37 | 11 | 77.083 |
| SAMB-057 | 48 | 41 | 7 | 85.417 |
| SAMB-058 | 48 | 40 | 8 | 83.333 |
| SAMB-059 | 48 | 44 | 4 | 91.667 |
| SAMB-060 | 48 | 43 | 5 | 89.583 |
| SAMB-061 | 48 | 46 | 2 | 95.833 |
| SAMB-062 | 48 | 40 | 8 | 83.333 |
| SAMB-063 | 48 | 42 | 6 | 87.500 |
| SAMB-064 | 48 | 38 | 10 | 79.167 |
| SAMB-065 | 48 | 39 | 9 | 81.250 |
| SAMB-066 | 48 | 43 | 5 | 89.583 |
| SAMB-067 | 48 | 45 | 3 | 93.750 |
| SAMB-068 | 48 | 45 | 3 | 93.750 |
| SAMB-069 | 48 | 44 | 4 | 91.667 |
| SAMB-070 | 48 | 44 | 4 | 91.667 |
| SAMB-071 | 48 | 43 | 5 | 89.583 |
| SAMB-072 | 48 | 46 | 2 | 95.833 |
| SAMB-073 | 48 | 48 | 0 | 100.000 |
| SAMB-074 | 48 | 43 | 5 | 89.583 |
| SAMB-075 | 48 | 41 | 7 | 85.417 |
| SAMB-076 | 48 | 45 | 3 | 93.750 |
| SAMB-077 | 48 | 40 | 8 | 83.333 |
| SAMB-078 | 48 | 42 | 6 | 87.500 |
| SAMB-079 | 48 | 42 | 6 | 87.500 |
| SAMB-080 | 48 | 40 | 8 | 83.333 |
| SAMB-081 | 48 | 39 | 9 | 81.250 |
| SAMB-082 | 48 | 41 | 7 | 85.417 |
| SAMB-083 | 48 | 44 | 4 | 91.667 |
| SAMB-084 | 48 | 45 | 3 | 93.750 |
| SAMB-085 | 48 | 46 | 2 | 95.833 |
| SAMB-086 | 48 | 37 | 11 | 77.083 |
| SAMB-087 | 48 | 37 | 11 | 77.083 |
| SAMB-088 | 48 | 40 | 8 | 83.333 |
| SAMB-089 | 48 | 39 | 9 | 81.250 |
| SAMB-090 | 48 | 46 | 2 | 95.833 |
| SAMB-091 | 48 | 46 | 2 | 95.833 |
| SAMB-092 | 48 | 39 | 9 | 81.250 |
| SAMB-093 | 48 | 39 | 9 | 81.250 |
| SAMB-094 | 48 | 44 | 4 | 91.667 |
| SAMB-095 | 48 | 46 | 2 | 95.833 |
| SAMB-096 | 48 | 44 | 4 | 91.667 |
| SAMB-097 | 48 | 37 | 11 | 77.083 |
| SAMB-098 | 48 | 44 | 4 | 91.667 |
| SAMB-099 | 48 | 37 | 11 | 77.083 |
| SAMB-100 | 48 | 41 | 7 | 85.417 |

Raw bit accuracy was calculated from 48 non-FM evaluated positions, comprising 45 graph-payload bits and three parity bits. The FM site P0(0,0) was used for orientation registration and was excluded from the accuracy calculation.

**Supplementary Table 6 | Register structure screening and test-set inclusion by batch.**

| **Preparation batch** | **Imaging batch** | **Examined,** $\boldsymbol{n}$ | **Incomplete,** $\boldsymbol{n}$ | **Deformed/stacked,** $\boldsymbol{n}$ | **Unidentifiable FM,** $\boldsymbol{n}$ | **Readable,** $\boldsymbol{n}$ **(% of examined)** | **Test set,** $\boldsymbol{n}$ |
| --- | --- | --- | --- | --- | --- | --- | --- |
| **P1** | I1 | 76 | 27 | 7 | 15 | 27 (35.53%) | 18 |
| **P2** | I2 | 104 | 26 | 11 | 32 | 35 (33.65%) | 19 |
| **P3** | I3 | 133 | 41 | 28 | 26 | 38 (28.57%) | 19 |
| **Total** | — | 313 | 94 | 46 | 73 | 100 (31.95%) | 56 |

Note: Exclusion categories were mutually exclusive. Structures were screened sequentially for incomplete assembly, severe deformation or stacking, and an unidentifiable fiducial marker (FM). Each structure was counted only once, in the first applicable exclusion category. Structures passing all three criteria were classified as readable. The training/development subset and the randomly selected test subset were non-overlapping subsets of the readable structures (44 + 56 = 100).

**Supplementary Table 7 | Batch-resolved readout performance of a randomly selected test subset**

| **Batch pair** | **Test structures,** $\boldsymbol{n}$ | **Raw bit accuracy** $\mathbf{(}\boldsymbol{TP}\boldsymbol{+}\boldsymbol{TN}\mathbf{)}\boldsymbol{/N}$ **(%)** | **False-positive rate *FP***$\boldsymbol{/}\boldsymbol{N}_{\boldsymbol{0}}$**(%)** | **False-negative rate *FN***$\boldsymbol{/}\boldsymbol{N}_{\boldsymbol{1}}$**(%)** |
| --- | --- | --- | --- | --- |
| **P1/I1** | 18 | 674/810 (83.21%) | 47/540 (8.70%) | 89/270 (32.96%) |
| **P2/I2** | 19 | 764/855 (89.36%) | 34/570 (5.96%) | 57/285 (20.00%) |
| **P3/I3** | 19 | 749/855 (87.60%) | 42/570 (7.37%) | 64/285 (22.46%) |
| **Overall** | 56 | 2187/2520 (86.79%) | 123/1680 (7.32%) | 210/840 (25.00%) |

Note: Each SAMB contained 45 evaluated graph-bit sites, comprising 30 expected 0-bit sites and 15 expected 1-bit sites. The FM site and three parity-bit sites were excluded from the analysis. $\boldsymbol{N}$, $\boldsymbol{N}_{\boldsymbol{0}}$, and $\boldsymbol{N}_{\boldsymbol{1}}$ denote the total number of evaluated sites, expected 0-bit sites, and expected 1-bit sites, respectively. FP and FN denote a 0-bit misclassified as 1 and a 1-bit misclassified as 0, respectively.

**Supplementary References**

1	Dickinson, G. D. *et al.* An alternative approach to nucleic acid memory. *Nat Commun* **12**, 2371 (2021). <https://doi.org/10.1038/s41467-021-22277-y>

2	Pan, C. *et al.* Rewritable two-dimensional DNA-based data storage with machine learning reconstruction. *Nat Commun* **13**, 2984 (2022). <https://doi.org/10.1038/s41467-022-30140-x>

3	Zhang, Y. *et al.* DNA origami cryptography for secure communication. *Nat Commun* **10**, 5469 (2019). <https://doi.org/10.1038/s41467-019-13517-3>

4	Wisna, G. B. M. *et al.* High-speed 3D DNA PAINT and unsupervised clustering for unlocking 3D DNA origami cryptography. *Nat Commun* **16**, 11514 (2025). <https://doi.org/10.1038/s41467-025-66338-y>

5	Zhang, C., Xie, M., Wang, L., Fan, C. & Chao, J. Linked data storage using DNA origami nanostructures. *Nat Commun* **16**, 11422 (2025). <https://doi.org/10.1038/s41467-025-66274-x>
